# Molecular dissection of the domain architecture and catalytic activities of human PrimPol

**DOI:** 10.1093/nar/gku214

**Published:** 2014-03-20

**Authors:** Benjamin A. Keen, Stanislaw K. Jozwiakowski, Laura J. Bailey, Julie Bianchi, Aidan J. Doherty

**Affiliations:** Genome Damage and Stability Centre, University of Sussex, Brighton, BN1 9RQ, UK

## Abstract

PrimPol is a primase–polymerase involved in nuclear and mitochondrial DNA replication in eukaryotic cells. Although PrimPol is predicted to possess an archaeo-eukaryotic primase and a UL52-like zinc finger domain, the role of these domains has not been established. Here, we report that the proposed zinc finger domain of human PrimPol binds zinc ions and is essential for maintaining primase activity. Although apparently dispensable for its polymerase activity, the zinc finger also regulates the processivity and fidelity of PrimPol's extension activities. When the zinc finger is disrupted, PrimPol becomes more promutagenic, has an altered translesion synthesis spectrum and is capable of faithfully bypassing cyclobutane pyrimidine dimer photolesions. PrimPol's polymerase domain binds to both single- and double-stranded DNA, whilst the zinc finger domain binds only to single-stranded DNA. We additionally report that although PrimPol's primase activity is required to restore wild-type replication fork rates in irradiated *PrimPol^−/−^* cells, polymerase activity is sufficient to maintain regular replisome progression in unperturbed cells. Together, these findings provide the first analysis of the molecular architecture of PrimPol, describing the activities associated with, and interplay between, its functional domains and defining the requirement for its primase and polymerase activities during nuclear DNA replication.

## INTRODUCTION

DNA replication is an essential biological process, indispensable for the existence of life. DNA replication systems rely on a semi-conservative mode of replication where the initiation of DNA synthesis requires a free 3′ hydroxyl group to which additional nucleotides are subsequently added by replicative polymerases (1). Genome replication starts with DNA template-dependent synthesis of short RNA primers that are further extended with deoxynucleotides by the replication machinery. This initial step in DNA replication is often defined as *de novo* primer synthesis and is catalysed by specialised DNA polymerases known as primases. Based on their structural topology, these enzymes can be classified into archaeo-eukaryotic primases (AEPs) or DnaG-like prokaryotic primases (2,3).

Until recently, the Pol α-associated DNA primase small subunit (PriS) that is responsible for *de novo* polymer synthesis through the production of RNA primers was considered to be the sole AEP superfamily member present in eukaryotes (1). However, bioinformatic analysis identified the existence of an additional uncharacterized DNA primase in eukaryotes called PrimPol (CCDC111 or FLJ33167) (3–7), which belongs to the ‘NCLDV-herpesvirus clade’ of viral AEPs.

Recent studies have reported that PrimPol is a DNA primase (4–7), with the ability to synthesise primers using either ribonucleotides (NTPs) or deoxyribonucleotides (dNTPs), preferring to make DNA primers. In addition, the enzyme possesses robust template-dependent DNA polymerase activity (4–7). PrimPol is present in both the nucleus (4–6) and mitochondria (7) of eukaryotic cells. The enzyme localises to nuclear chromatin during replication (4–6) and this recruitment is more pronounced after treatment with damaging agents (e.g. ultraviolet light; UV) or replication stalling drugs (e.g. hydroxyurea) (4). UV irradiation can induce the covalent linkage of adjacent pyrimidines leading to the formation of cyclobutane pyrimidine dimers (CPDs) and pyrimidine (6–4) pyrimidone photoproducts ((6–4)PPs). These helix-distorting lesions interfere with major biological processes, including DNA replication and transcription (8). PrimPol can perform translesion synthesis (TLS) bypass of the highly distorting (6–4)PPs but is also involved in replication through oxidative lesions, including 8-oxoguanine (8-oxoG) (4,5,7). Deletion of PrimPol (*PrimPol^−/−^*) induced replication fork slowing, which was much more pronounced when cells were UV irradiated (4). Knockout cells also exhibited increased formation of chromosomal breaks, particularly after aphidicolin treatment. This study also reported that PrimPol is not epistatic with the Pol η-dependant CPD-bypass pathway and therefore appears to form an independent pathway required for the bypass of UV, and other lesions, during replication (4,5).

**Figure 1.**
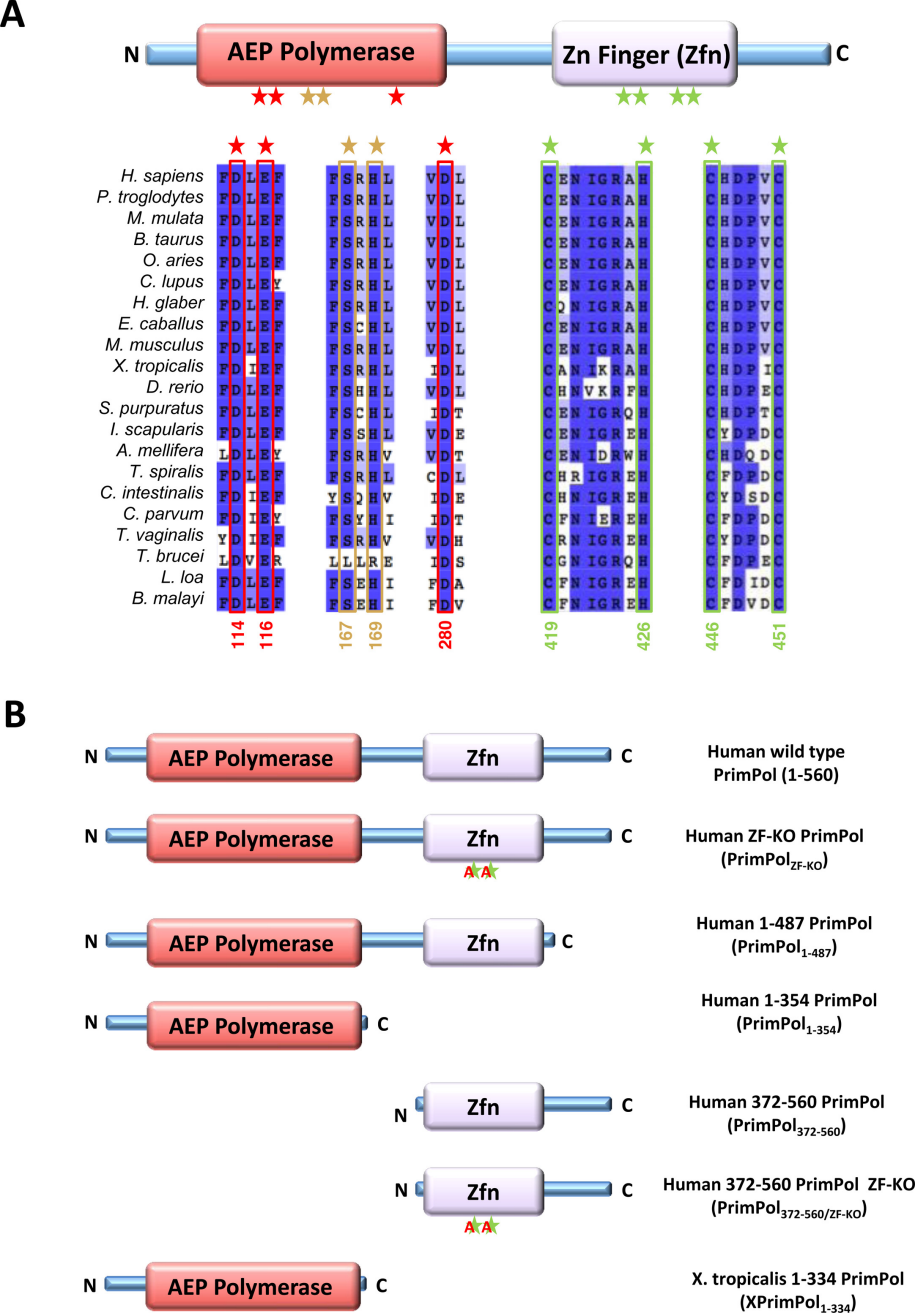
Domain architecture of eukaryotic PrimPol. (**A**) PrimPol is composed of an AEP primase–polymerase and a Zn^2+^ finger (Zfn) domain. The AEP domain contains three conserved catalytic motifs (I–III). The first motif (motif I, the first two red stars) is the DxE motif that, along with the conserved D in the third motif (motif III, third red star), forms the catalytic triad that coordinates divalent metal ions essential in the synthesis of oligonucleotide chains. The second motif (motif II, two light-orange stars) is a conserved SxH motif that is required for the coordination of the incoming nucleotide. The zinc finger domain contains a canonical C–H–C–C motif that coordinates a zinc ion and stabilises the anti-parallel β-sheet and α helix structure. The numbers below the conserved residues denote their positions in human PrimPol. (**B**) A number of human PrimPol constructs were produced to analyse the domain architecture of PrimPol. A Zfn knockout mutant was produced by mutating the first conserved cysteine and histidine residues that coordinate the zinc ion (C419A and H426A, respectively). We constructed a number of truncation mutants based on secondary structure predictions. PrimPol_1–487_ lacks the C-terminus region downstream of the Zfn domain. PrimPol_1–354_ deletion mutant contains the polymerase domain but lacks the Zfn domain. PrimPol_372–560_ possesses the zinc finger domain but lacks the polymerase domain. PrimPol_1–354/ZF-KO_ contains only the zinc finger domain but the C419A and H426A are mutated. A 1–334 deletion mutant in *Xenopus tropicalis* (XPrimPol_1–334_) was additionally constructed that is equivalent to PrimPol_1–354_.

Comparative analysis of the amino acid sequence of eukaryotic PrimPols identified the presence of two distinctive domains (3), an enzymatic AEP polymerase and a UL52-like zinc finger (Zfn) domain (Figure 1A). Mutation of the conserved zinc-chelating cysteine residues in the UL52 domain of herpes simplex virus type 1 resulted in a severe reduction in DNA binding (8,9). The AEP polymerase domain contains the three signature catalytic motifs. A highly conserved DxE motif (motif I), together with an aspartic acid residue from motif III, forms the divalent metal binding site. Motif II (SxH) forms part of the putative nucleotide binding motif. Mutagenesis of these motifs abolishes the catalytic activities of these polymerases (4,5,7,10,11).

In this study, we have dissected the molecular architecture of human PrimPol to define the activities associated with its two major functional domains. We demonstrate that the zinc finger domain is crucial for its primase activity. PrimPol also has DNA template-dependent DNA polymerase activities and this bifunctional enzymatic activity is reminiscent of archaeal replicative primases (11–14). Although PrimPol's polymerase activities appeared, initially, to be largely independent of the zinc finger domain, our data suggests that the zinc finger domain plays important roles in the processivity and fidelity of DNA synthesis. Additionally, we demonstrate that the zinc finger domain has regulatory roles for both DNA/RNA primase and TLS activities. We also report that a catalytically active fragment of human PrimPol, containing only the AEP domain (PrimPol_1–354_), catalyzes TLS bypass of both major UV-induced DNA lesions, CPDs and (6–4)PPs. Analysis of the DNA binding affinities of catalytically active PrimPol_1–354_ and the C-terminal UL52-like domains established that the enzymatic domain can bind both single-stranded (ss) and double-stranded (ds) DNA, whilst the zinc finger domain can only bind to ss DNA. Finally, we report that although PrimPol's polymerase activity is sufficient to maintain wild-type replication fork rates in unperturbed *PrimPol^−/−^* cells, primase activity is requisite for normal replication in UV-treated cells. Together, these findings provide the first molecular insights into the domain architecture of human PrimPol, defining the activities associated with its major functional modules and delineating the requirement for its specific catalytic activities during perturbed and unperturbed DNA replication.

## MATERIALS AND METHODS

### Construction of human PrimPol mutants

Human PrimPol was cloned as described previously (4). A number of PrimPol mutants were constructed by polymerase chain reaction (Figure 1B) and the primers used can be found in the supplementary data (Supplementary Table S1). *Xenopus tropicalis* PrimPol cDNA was sub-cloned into pET28a (Novagen) using BamHI and XhoI restriction sites. Subsequently, a 1–334 C-terminal deletion (XPrimPol_1–334_) mutant was also constructed (primers in Supplementary Table S1). Additionally, we constructed a PrimPol_AXA_ (catalytically null) mutant that was cloned and purified, as previously described (4).

### Expression and purification of recombinant PrimPol proteins

Wild-type PrimPol and PrimPol_1–487_ were expressed in *Escherichia coli* BL21(pLysS) cells overnight at 16°C, as described previously (4). PrimPol_1–354_, PrimPol_372–560_ and XPrimPol_1–334_ were expressed overnight at 25°C. All of the proteins were then purified by the same protocol, except where otherwise noted. Cells were pelleted and resuspended in buffer A (50 mM Tris–HCl (pH 7.5), 200 mM NaCl, 30 mM imidazole, 10% (v/v) glycerol, 17 μg/ml PMSF, 34 μg/ml benzamidine) supplemented with 0.5% IGEPAL. Cells were disrupted by sonication and proteins isolated by centrifugation. All proteins were purified by affinity chromatography using a 25 ml Ni^2+^-NTA agarose (Qiagen) column equilibrated with buffer A and eluted with buffer B (as A, with 300 mM imidazole). The eluted protein-containing fractions were then diluted 1 in 10 in buffer C (50 mM Tris–HCl (pH 7.5), 10% (v/v) glycerol) and separated by charge by affinity exchange chromatography on a 5 ml HiTrap Heparin HP column (GE Healthcare) equilibrated with buffer C. PrimPol_372–560_ was purified on a 5 ml HiTrap Q HP (GE Healthcare) column equilibrated with buffer C. All proteins were subject to gradient elution with buffer D (as C, with 2M NaCl) and protein-containing fractions were purified by size exclusion chromatography on a Superdex S-75 analytical gel-filtration column (GE Healthcare) equilibrated in buffer E (50 mM Tris–HCl (pH 7.5), 300 mM NaCl, 10% (v/v) glycerol). Protein concentrations were determined by absorption spectra at 280 nm.

### DNA primase assays

The sequences of the oligonucleotides used in the primase assays are sequences 1–4 in Supplementary Table S2. The non-radioactive primase assay was performed in three subsequent steps. Typically detection of primase activity was started from incubation of 1μM of the enzyme to be tested in 20 μl reaction volume containing 500 nM homopolymeric ss DNA templates with a biotin modification at the 5′ end (see sequences 1–4 in Supplementary Table S2), 500 μM rNTPs (Invitrogen) or 500 μM dNTPs (Roche), 10 mM Bis-Tris-Propane-HCl (pH 7.0), 10 mM MgCl_2_, 50 mM NaCl. This primer synthesis reaction was carried out for 2 h at 37°C. Following the primer synthesis reaction, the reaction mixture was supplemented with 0.2 U of Klenow Taq (purified as in Engelke *et al.* (15)) and 15 μM FAM-6-dATP (Jena-Biosciences), incubated at 37°C for 45 min to allow fluorescent labelling of *de novo* synthesised primers. The primer synthesis/labelling enzymatic reactions were terminated by adding 450 μl of binding-washing (B-W) buffer (10 mM Tris-HCl (pH 8.0), 500 mM NaCl, 10 mM EDTA). The quenched reactions were subsequently added to 30 μl of streptavidin-coated beads (Invitrogen) and mixed on a spinning wheel for 1 h at 4°C. After binding the ss DNA templates, the suspensions were spun down briefly to sediment the beads. The supernatant was removed and the beads were washed three times with 1 ml B-W buffer. The beads were then suspended in 20 μl of the B-W buffer supplemented with equal volume of loading buffer (8 M Urea, 10 mM EDTA). The resulting sample was incubated at 95°C for 3 min in order to liberate the primers synthesised *de novo*. Subsequently, the samples were spun down briefly and 20 μl volume of each reaction was resolved by standard denaturing electrophoresis using a 15% (v/v) polyacrylamide gel matrix containing 7 M urea and 1× TBE buffer (100mM Tris, 100mM Boric Acid, 2mM EDTA). Typically the electrophoretic separation of primase assay products was performed at 850 V for ∼2 h. After electrophoresis gels were scanned for fluorescent signal detected by a Fujifilm FLA-5100 image reader.

### DNA primer extension assays

Hex-labelled DNA primers were annealed to oligomer templates (sequences in Supplementary Table S2). 34 nM of protein was incubated with 20 nM DNA, 10 mM Bis-Tris-Propane-HCl (pH 7.0), 10 mM MgCl_2_, 1 mM DTT and 200 μM dNTPs (Roche) to a final volume of 20 μl. In the case of single nucleotide incorporations 200 μM of each individual nucleotide adenosine, cytosine, guanosine or thymidine triphosphate (dATP, dCTP, dGTP or dTTP respectively) were added in lieu of dNTPs. The reactions were terminated by the addition of 2× stop buffer (95% formamide, 0.09% xylene cyanol, 0.05% bromophenol blue, 200 nM competitor oligonucleotide) and boiled at 95°C for 5 min. Samples were resolved by electrophoresis on a 15% (v/v) polyacrylamide gel containing 7 M urea and 1× TBE buffer at 850 V for 2.5 h in 1× TBE buffer. Fluorescently labelled DNA oligomers were detected by scanning using a Fujifilm FLA-5100 image reader.

### Terminal transferase assays

The terminal transferase capability of wild-type PrimPol and its variants was studied using three types of synthetic DNA substrates: ds DNA (sequence 6 annealed to sequence 7 from Supplementary Table S2) and a primer template containing both single-stranded and double-stranded DNA interfaces (sequence 5 annealed to sequence 6; Supplementary Table S2). Typical reactions were performed in 20 μl volume containing 10 mM Bis-Tris-Propane-HCl (pH 7.0), 10 mM NaCl, 10 mM MgCl_2_, 1 mM MnCl_2_, 1 mM DTT, 20 nM DNA substrate, 200 μM dNTPs (Roche), with 100 nM recombinant human PrimPol or its variants. All reactions were incubated at 37°C for 30 min, reactions were quenched with addition of 2× stop buffer (95% formamide, 0.09% xylene cyanol, 0.05% bromophenol blue, 200 nM competitor oligonucleotide) and boiled at 95°C for 5 min. Samples were resolved by electrophoresis as described for the primer extension assays.

### DNA extension processivity assays

Full-length and PrimPol_1–354_ constructs were analysed for processivity as described previously (16). PrimPol (100 nM) was preincubated at 37°C with 60 nM substrate DNA (sequence 5 annealed to sequence 6 from Supplementary Table S2), 10 mM Bis-Tris-Propane-HCl (pH 7.0), 10 mM MgCl_2_ and 1 mM DTT for 30 min. Reactions were initiated by adding dNTPs and excess of sonicated herring sperm DNA (1 mg/ml) as an enzyme trap. Reactions were quenched after time points of 15, 30, 60, 120 and 360s with addition of 2× stop buffer (95% formamide, 0.09% xylene cyanol, 0.05% bromophenolblue, 200 nM competitor oligonucleotide) and boiled at 95°C for 5 min. Samples were resolved by electrophoresis as described for the primer extension assays. To test the effectiveness of the trap, each of the enzymes were also preincubated with excess of herring sperm DNA (1 mg/ml), as well as 40 nM DNA, 10 mM Bis-Tris-Propane-HCl (pH 7.0), 10 mM MgCl_2_ and 1 mM DTT. To ensure the trap did not disrupt the processivity of the enzyme, the experiment was also carried out with the Klenow fragment of Taq polymerase.

The band intensities at 360 s were measured using ImageQuant software (GE Healthcare). The percentage of active polymerases at a given position is given by Equation (1):

(1) \begin{eqnarray*} \%\;{\rm active\;polymerases\;at}\;n \\ = (I_n + I_{n + 1} + I_{n + 2 \ldots } ) \times 100\%/(I_1 + I_2 + I_3 \ldots ) \end{eqnarray*}

Where *I*_1_ is the intensity at position 1, *I*_n_ is the intensity at position *n* and so on.

### Electrophoretic mobility shift assays

Electrophoretic mobility shift assays (EMSAs) were carried out on two synthetic substrates: ss DNA (sequence 7 from Supplementary Table S2) and ds DNA (sequence 6 annealed to sequence 7 from Supplementary Table S2). Varying concentrations of the human PrimPol_1–354_ and PrimPol_372–560_ constructs were added to 40 nM DNA, 10 mM Bis-Tris-Propane-HCl (pH 7.0), 10 mM MgCl_2_ and 1.0 mM DTT to a final volume of 20 μl and incubated at 25°C for 60 min. The reactions were supplemented with 2 μl 25% (w/v) ficoll. Samples were resolved by electrophoresis on a 5% (v/v) polyacrylamide gel containing 0.5× TBE buffer at 150 V for 0.5 h, then 300 V for 2.5 h in 0.5× TBE buffer. Fluorescently labelled DNA oligomers were detected by scanning using a Fujifilm FLA-5100 image reader.

### Inductively coupled plasma mass spectrometry

The concentrations of zinc ions in protein samples were determined using inductively coupled plasma mass spectrometry (ICP-MS). For this analysis of human PrimPol, the proteins were gel filtered in zinc-free solutions to minimise the concentration of zinc ions that were not chelated by protein. Quantitative calibrations were made using standards prepared from zinc solutions containing 0, 100, 500 and 1000 ng/ml. Each sample was measured in triplicate and background measurements of buffer without protein were determined and subtracted from the appropriate readings. A 2% nitric acid wash was performed between standards and samples.

### PrimPol protein denaturation assays

The full-length and PrimPol_1–487_ constructs were both dialysed into a buffer of 200 mM NaF, 15 mM Tris-HCl (pH 7.5). 45 μl of 1 μM protein solution was added to 15 μl of SYPRO Orange, resulting in a final protein concentration of 0.75 μM. A control of 45 μl buffer was also added to 15 μl of SYPRO Orange. 20 μl of each sample was aliquoted in triplicate into a 96-well plate. Protein melting experiments were carried out using the LightCycler 480 System II (Roche). The instrument was configured with a detection format of 465 nm as the wavelength of excitation and 580 nm as the emission wavelength to detect SYPRO Orange-specific signal. Denaturation curve fluorescent signal was acquired within a range of 20–80°C using a ramping rate of 0.03°C s^−1^ and an acquisition of 20 data points per degree celsius. Melting temperatures (*T*_m_) were determined through the measurement of the lowest point of the negative differential of the denaturation curve. Data was corrected for the background signal of the buffer conditions and presented as units of fluorescence with respect to temperature ± 1 SD.

### Complementation and survival assays in *PrimPol^−/−^* avian cells

DT40 cells were grown at 39°C in RPMI 1640 medium supplemented with 10 μM β-mercaptoethanol, penicillin, streptomycin, 10% foetal calf serum and 1% chicken serum (Sigma). DT40 cells deleted of PrimPol made previously (4) were stably complemented with wild-type and truncated or mutated forms of human PrimPol cloned into the pCI-neo vector by electroporation as described previously (4). Stable expressing cells were selected using 2 mg/ml G418 (Sigma) and confirmed by western blot. DNA fibre analysis was carried out as described previously (4). Fibre analysis was carried out in triplicate (*n* = 3).

## RESULTS

### Zinc finger domain of PrimPol coordinates a zinc ion

The C-terminal domain of human PrimPol contains a highly conserved UL52-like Cys-His-Cys-Cys (CHC2) motif, predicted to fold into a functional zinc finger (Zfn) motif. (Figure 1A) To establish if this is a *bona fide* zinc-binding motif, we produced C-terminal truncations in PrimPol containing either an intact (PrimPol_372–560_) or mutated (PrimPol_372–560/ZF-KO_) Zfn motif. PrimPol_372–560/ZF-KO_ has the first two residues of the Zfn motif mutated to alanine. The proteins were gel filtered in the absence of zinc and concentrations of zinc were then measured using ICP-MS (Supplementary Table S3). The ICP-MS analysis revealed that the concentration of the zinc in the UL52-like domain represented an occupancy of 80.38%, suggesting that a small fraction of the protein is either unfolded or has another divalent metal ion chelated. In contrast, the zinc finger knockout had an occupancy of 3.90%, suggesting that the first two cysteine and histidine residues of this motif are required for the coordination of zinc. We also measured the zinc occupancy in the polymerase domain (PrimPol_1–354_) after purification in the absence of zinc and the occupancy in this case was 1.57%. The detection of low levels of zinc in the polymerase domain is to be expected as it contains divalent cation-binding motifs. These motifs generally bind magnesium or manganese, which have respective ionic radii of 86 and 81 pm, but could conceivably bind zinc, with a comparable ionic radius of 88 pm (17).

### PrimPol's zinc finger domain is essential for primase activity

Previously, we and others have shown that PrimPol has DNA primase activity *in vitro* (4–7), preferentially catalysing synthesis of primers composed of DNA. Previous studies on the viral HSV1 helicase/primase complex showed that mutation of key residues in the UL52 zinc-binding domain of this enzyme resulted in loss of primase activity (8,9). Alignment of human PrimPol with other eukaryotic orthologues identified the conserved residues (C419, H426, C446 and C451; Figure 1A) predicted to coordinate a zinc ion. We prepared a zinc finger knock-out enzyme carrying C419A and H426A point mutations (PrimPol_ZF-KO_) (Figure 1B).

**Figure 2.**
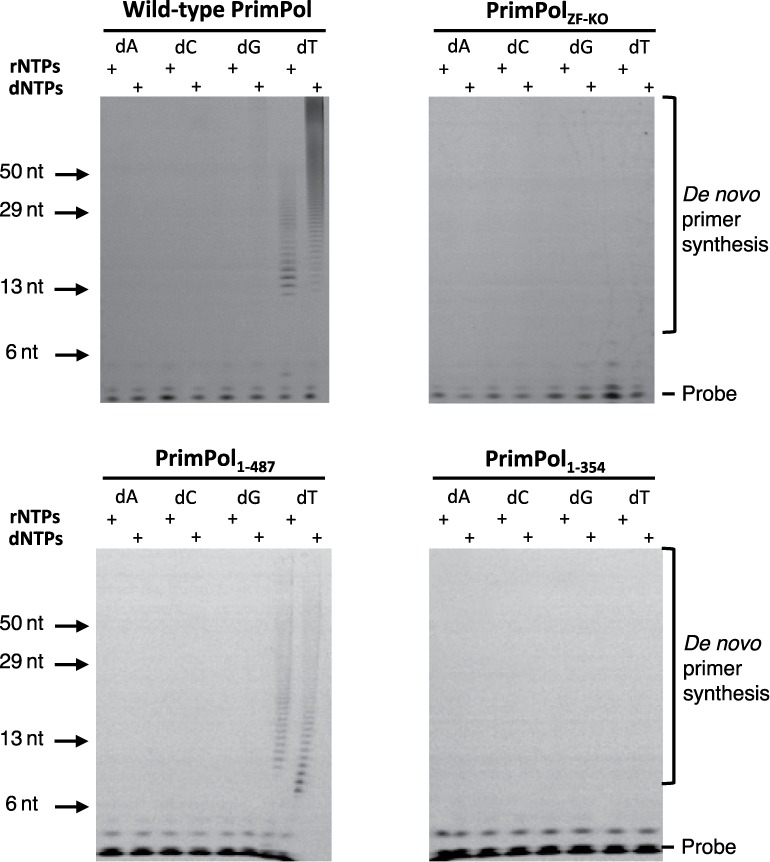
Primase activity of human PrimPol. (**A**) Human PrimPol has primase activity and can produce *de novo* primers using rNTPs and dNTPs opposite a poly(dT) template. (**B**) PrimPol_ZF-KO_ lacks *de novo* primer synthesis activity, suggesting that an intact zinc finger is required for primase activity. (**C**) PrimPol_1–487_ also has primase activity similar to the wild-type PrimPol. The unstructured region that is downstream of the zinc finger is therefore not required for primase activity. (**D**) PrimPol_1–354_ has no primase activity, which indicates that PrimPol requires a functional zinc finger for primer synthesis.

As previously observed (4,5), wild-type PrimPol exhibited primase activity on a 60-mer poly-dT ss DNA template, however, PrimPol_ZF-KO_ showed no detectable primase activity (Figure 2). Furthermore, we analysed the primase activity of two deletion mutants, PrimPol_1–487_ and PrimPol_1–354_, which were constructed to determine whether elimination of primase activity resulted from a structural change brought about by the point mutations or whether the zinc-finger domain is necessary for primase activity. As expected, a deletion mutant lacking the entire zinc finger (PrimPol_1–354_) showed no detectable primase activity (Figure 2). However, PrimPol_1–487_, containing the entire zinc-finger domain but lacking the smaller C-terminal portion, predicted to be disordered, was an active DNA primase (Figure 2). Notably, the observed primase activity of PrimPol_1–487_ was lower than the activity observed for the parental enzyme, possibly as this truncation results in partial loss of UL52 domain functionality. Together, our data provide experimental evidence that the zinc-finger domain, but not the disordered region proximal to this domain, is essential for the primase activity of human PrimPol.

### PrimPol's DNA polymerase activity is modulated by the zinc finger domain

**Figure 3.**
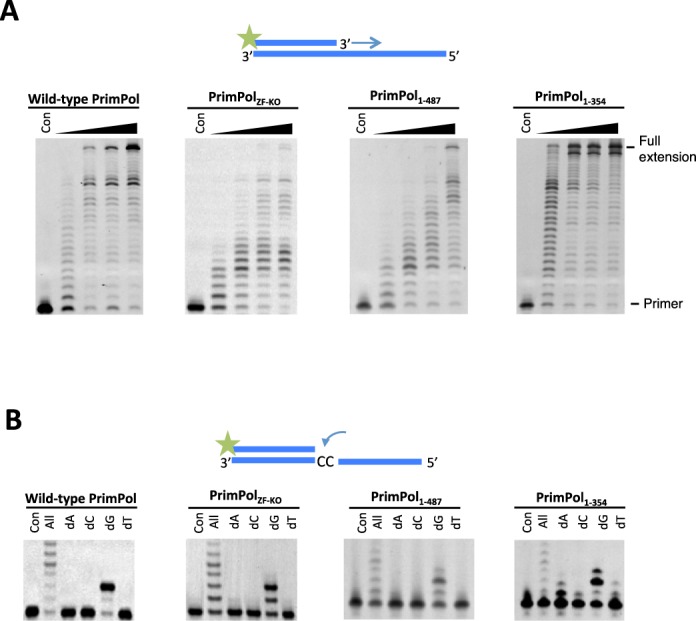
Polymerase activity and fidelity of human PrimPol. (**A**) Human PrimPol was incubated with dNTPs and substrate at 1, 3, 5 and 30 min time points. PrimPol was proficient at extending an undamaged oligonucleotide template using dNTPs. Human PrimPol did not require an intact zinc finger in order to carry out primer extension, as evidenced by the extension of primers by PrimPol_ZF-KO_ and PrimPol_1–354_. PrimPol_1–487_ that lacked the unstructured C-terminus of the protein was also polymerase proficient. PrimPol_1–354_ exhibited a higher rate of polymerase activity compared to the other constructs. (**B**) Incorporation of nucleotides opposite two templating cytosine bases. PrimPol was incubated for 5 min with the DNA substrate and each of the dNTPs. All four of these PrimPol constructs inserted two guanine nucleotides opposite two cytosines in Watson–Crick base-pairing manner. PrimPol_1–354_ could additionally incorporate a single adenine opposite the first cytosine.

As the zinc-finger domain is required for the priming activity of human PrimPol, we also evaluated the importance of this functional module in the context of PrimPol's DNA polymerase activity. To address this, we employed standard primer extension assays using unmodified synthetic primer templates and dNTPs. We compared the relative DNA polymerase activities of four different variants of human PrimPol including the wild-type enzyme, PrimPol_ZF-KO_, PrimPol_1–487_ and PrimPol_1–354_ (Figure 1B; Table 1A). Although all tested variants of PrimPol were proficient at DNA template-dependent DNA synthesis, we observed variation in the relative specific polymerase activity, processivity and fidelity among these enzymes (Figure 3A). PrimPol_ZF-KO_ showed a pronounced decrease in specific DNA polymerase activity. In contrast, PrimPol_1–354_ exhibited both increased relative DNA processivity and extension activities. Despite variations in specific DNA polymerase activities across the variants of human PrimPol tested, the zinc-finger module does not appear to be essential for DNA polymerase activity of this enzyme. However, a regulatory role in catalysis for this functional module cannot be excluded.

**Table 1.**
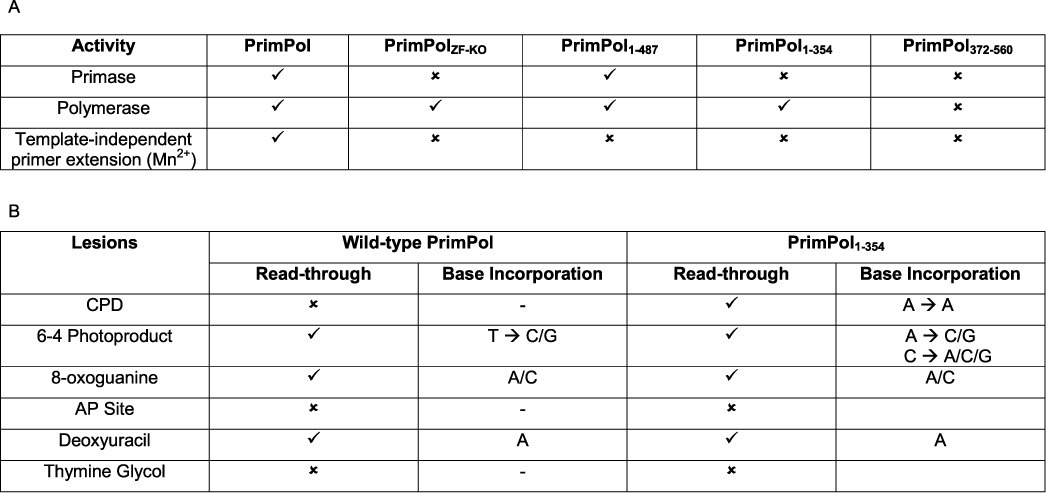
Extension, TLS and fidelity activities associated with full-length and mutated forms of human PrimPol

**Summary of the DNA synthesis activities of wild-type and mutant PrimPol.** (**A**) PrimPol exhibits primase activity, polymerase activity and, in the presence of manganese, template-independent primer extension. Whilst the polymerase activity and template-independent primer extension activity appear to reside in the AEP polymerase domain alone, the primase activity requires the associated zinc finger. (**B**) PrimPol can read through a number of common DNA lesions and we have determined some of the lesions PrimPol can read through and which bases are incorporated opposite these lesions. As the UV lesions span two adjacent bases, a ‘→’ is used to separate the bases incorporated opposite the first and second damaged bases.

Consequently, we next examined the potential regulatory role of this functional module. In particular, we tested the fidelity of incorporation of each of the four single deoxynucleotides opposite two templating cytosines (Figure 3B). Wild-type PrimPol, PrimPol_ZF-KO_ and PrimPol_1–487_ all incorporated two correct incoming guanine nucleotides opposite the templating cytosines at the N+1 and N+2 positions. In contrast, PrimPol_1–354_ that lacks the entire zinc finger domain showed a significantly reduced ability to accurately select the incoming nucleotide. In particular, we observed significant mis-incorporation of adenine nucleotides opposite templating cytosine at the N+1 position and mis-incorporation of another guanine nucleotide opposite a templating adenine at N+3. This observation suggests the potential importance of the Zfn domain in influencing the fidelity of PrimPol's polymerase activity. PrimPol cannot incorporate NTPs during primer extension *in vitro* and mutation of the Zfn domain did not alter this preference (unpublished data). This is in contrast to the AEP domain of LigD, which can extend primers using both dNTPs and NTPs (18,19).

### PrimPol exhibits template-independent primer extension activity in the presence of manganese

**Figure 4.**
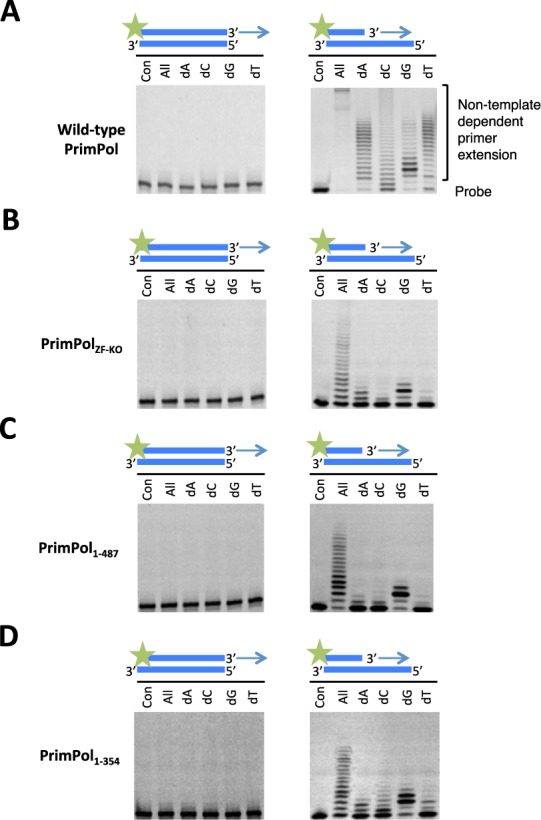
Template-independent extension in the presence of manganese. Human PrimPol was incubated for 30 min with DNA substrate and each of the dNTPs in the presence of manganese. (**A**) Wild-type PrimPol was unable to extend from a ds DNA template with a blunt end, but could extend from a primer annealed to an overhanging template, even synthesising long tracts of homopolymers. (**B**) PrimPol_ZF-KO_ could extend from an overhanging DNA template in the presence of manganese and, consistent with the wild-type, did not extend from a ds DNA substrate. The incorporation of 1 or 2 nucleotides of guanine or adenine opposite an overhanging template suggests that PrimPol_ZF-KO_ incorporates in a low-fidelity template-dependent manner when incubated with overhanging DNA. (**C**) PrimPol_1–487_ exhibited a highly similar terminal transferase activity spectrum to the PrimPol_ZF-KO_. In the presence of manganese, it incorporated bases opposite an overhang in a low fidelity, template-dependent manner. (**D**) PrimPol_1–354_ also exhibited low fidelity extension of a primer annealed to an overhanging template in the presence of manganese.

The flexible extension activities reported previously for Non homologous end-joining (NHEJ) AEP polymerases include limited template-independent terminal transferase activity on both ss DNA and blunt-ended ds DNA (18,19). Such activities are often stimulated by the presence of manganese ions, which can accelerate the rate of DNA synthesis and, as a result, lower the overall fidelity of this process. We therefore tested if human PrimPol also possesses terminal transferase activity in the presence of manganese. Wild type, as well as all three variants, of human PrimPol showed no detectable terminal transferase activity when tested on a blunt ended ds DNA substrate (Figure 4, left panels). However, PrimPol's DNA synthesis activity appeared to be significantly increased when primer extension assays were performed in the presence of dNTPs (all) and manganese (Figure 4A, right panel). To investigate if this increased activity resulted from altered fidelity, we repeated the assays with each of the dNTPs and observed that PrimPol catalysed multiple extension of the primer even in the presence of a single nucleotide pool. This promiscuous DNA synthesis was most efficient when enzyme was utilising dATP or dTTP. In both cases, extension of primers by up to ∼20 nucleotides was observed. Notably, this phenomenon was not observed when magnesium was used instead of manganese. When a single incorporation of dCTP was investigated, we observed significantly slower extension rates but the enzyme was able to extend the primer to produce predicted full-length product. In contrast, incorporation of the correct incoming nucleotide (dGTP) resulted in least pronounced primer extensions up to 16 nucleotides.

When this experimental strategy was repeated to study potential promiscuous DNA polymerase activities in the PrimPol_ZF-KO_ and PrimPol_1–487_ variants, we observed minimal mis-incorporation of all four tested single nucleotides. Interestingly, when a catalytically active fragment (PrimPol_1–354_) was tested, we observed more efficient mis-incorporation compared to PrimPol_ZF-KO_ and PrimPol_1–487_ variants. However, PrimPol_1–354_ fragment was able to mis-incorporate between 1 and 2 incorrect incoming nucleotides. We are unable to conclude if this unique enzymatic activity of human PrimPol is significant for the biological functions of this enzyme *in vivo*. We speculate that, under the experimental conditions tested, the Zfn module allowed manganese-dependent primer extension resulting in the synthesis of DNA that is non-complementary to the template strand. This hypothetical scenario is only possible when the enzyme was able to bind to a single-stranded template strand of the DNA primer-template substrate. This interesting observation has raised questions regarding the mechanism by which PrimPol binds to and coordinates the primer-template substrate during DNA polymerisation.

### DNA binding activities of human PrimPol

**Figure 5.**
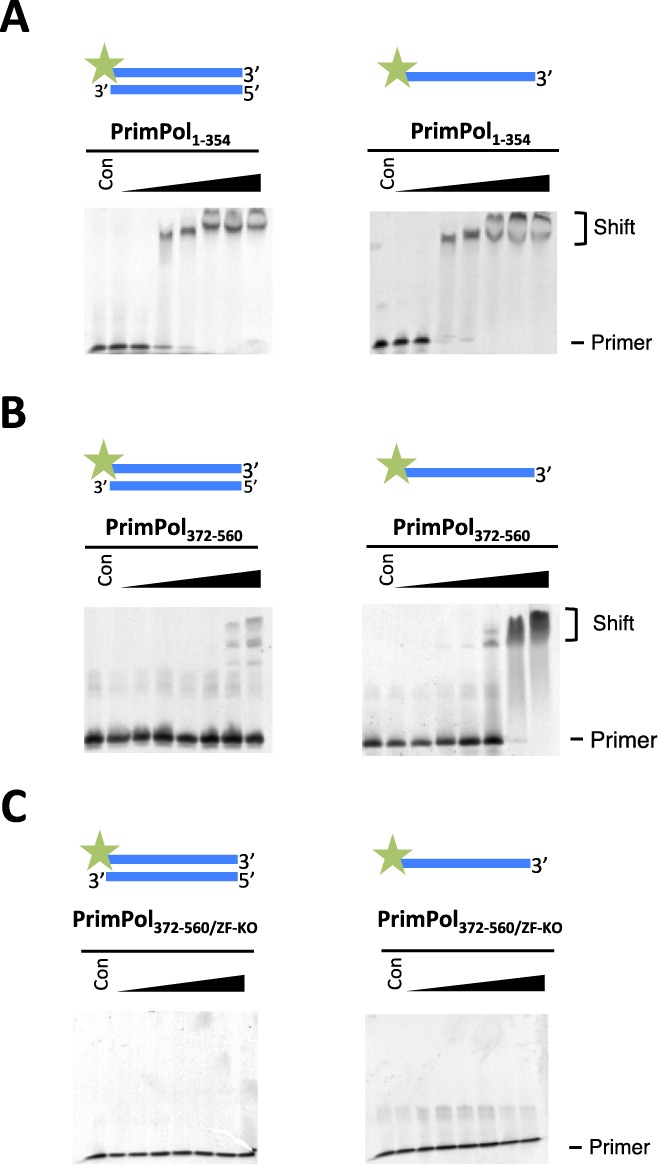
PrimPol's polymerase domain binds ss and ds DNA but the zinc finger domain binds only ss DNA. (**A**) PrimPol_1–354_ was tested for its ability to bind DNA by shift assays. PrimPol polymerase domain was incubated with 40 nM DNA at various protein concentrations (0.05, 0.1, 0.5, 1.0, 5.0, 10.0 and 20.0 μM) for 60 min at 25°C. The polymerase domain binds to ds DNA and ss DNA with approximately equal proficiency. Binding was evident at concentrations ∼0.5 μM but a complete shift was observed at 5.0 μM for each DNA substrate tested. (**B**) PrimPol_372–560_ (Zfn domain) was also assayed for binding to DNA activity. It was incubated with 40 nM DNA at various protein concentrations (0.05, 0.1, 0.5, 1.0, 5.0, 10.0 and 20.0 μM). A DNA shift was not observed with ds DNA substrate but a clear shift was evident with ss DNA, suggesting that the zinc finger binds single-stranded regions of DNA. (**C**) PrimPol_372–560/ZF-KO_, lacking a functional Zfn, was incubated with 40 nM DNA at the same protein concentrations and ss DNA binding activity was not observed.

Next, we analysed the binding of the AEP polymerase and Zfn domains of PrimPol to a number of DNA substrates to determine whether they bind specifically to ss or ds DNA. The Zfn domain is presumed to be a DNA-recognition domain, in addition to contributing to its primase activity, as previous studies demonstrated altered DNA binding activity associated with mutations in a related UL52 Zfn domain (8,20). EMSAs were performed to analyse the binding capabilities of the AEP polymerase (PrimPol_1–354_) and Zfn (PrimPol_372–560_) domains. PrimPol_1–354_ showed a similar binding capacity to ds and ss DNA, suggesting that the polymerase can bind both substrates (Figure 5A). The observed DNA binding specificity reflects the likely presence of two closely clustered binding sites allowing recognition of a primer-template junction, which is an essential requirement for all DNA polymerases. Observed binding values for both ss DNA and ds DNA were evident at a minimum of ∼500 nM DNA and a complete shift of the DNA was evident at 5 μM DNA. This relatively weak coordination of DNA substrates is consistent with previously postulated biological functions of human PrimPol (4–7).

Analogous EMSA experiments were also performed to analyse the DNA binding capacity of the Zfn domain (PrimPol_372–560_). In contrast with the AEP domain, PrimPol's Zfn domain was unable to bind ds DNA but did show specific binding to ss DNA (Figure 5B). This intriguing result provides experimental evidence supporting the potential importance of the Zfn module in recognition/coordination of the single-stranded template DNA downstream of the primer-template junction. The significance of this interaction will be discussed below. To determine if DNA binding was performed by the Zfn, we assayed a Zfn knockout domain (PrimPol_372–560/ZF-KO_) for ss DNA binding activity (Figure 5C) and observed an absence of binding with this mutant, confirming the importance of the Zfn motif for nucleic acid recognition.

### PrimPol's AEP domain possesses TLS activities

**Figure 6.**
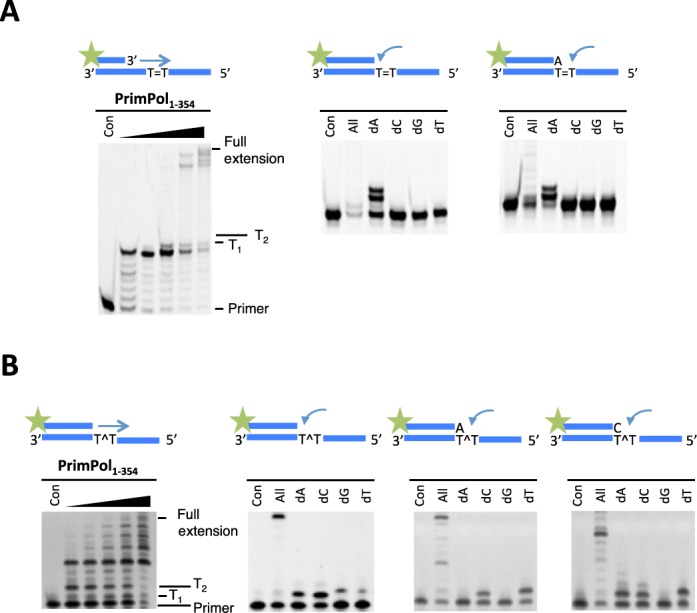
PrimPol_1–354_ can replicate through CPD and (6–4(PP)) lesions. (**A**) PrimPol_1–354_ was incubated with a primer-template substrate in which the template contained a CPD lesion downstream of the primer-template junction in the presence of dNTPs. Time points were taken between 0.5 and 60 min. PrimPol_1–354_ extends from the primer up to the CPD, before stalling, it will then added a base opposite the first thymine of the CPD and continue to extend until the end of the template (left panel). PrimPol_1–354_ was then incubated with each dNTP to test which nucleotide it incorporates opposite a CPD (right panels). PrimPol_1–354_ incorporated adenine opposite the first and second thymine of the CPD. (**B**) The polymerase domain of PrimPol can also perform TLS bypass of a 6–4(PP) lesion immediately downstream of the primer-template junction (left panel). PrimPol_1–354_ incorporates either an adenine or cytosine nucleotide opposite the first thymine of the 6–4(PP) (right panels). If an adenine is incorporated opposite the first thymine, a cytosine or thymine is then incorporated opposite the second. If a cytosine is incorporated opposite the first thymine, an adenine, cytosine or thymine will be incorporated opposite the second thymine of the 6–4(PP).

PrimPol has the capacity to bypass a number of replication stalling lesions, including UV ((6–4)PP) (4) and oxidative lesions (4,6). To address whether the TLS activities of PrimPol are independent of the zinc finger domain, we assayed the TLS activities of PrimPol_1–354_ on a range of templates containing a variety of DNA modifications (Table 1B). As full-length PrimPol appears unable to read through CPDs *in vitro*, we assayed PrimPol_1–354_ for TLS bypass of this common photolesion. Notably, although PrimPol_1–354_ slowed as it reached a CPD lesion, it showed the capacity to read through this bulky lesion (Figure 6A). In contrast, wild-type PrimPol cannot perform TLS opposite this lesion (4,5). Single nucleotide incorporation assays were employed to determine which bases are incorporated opposite CPDs, revealing that PrimPol performs error-free bypass of this photolesion (Figure 6A). A similar TLS activity was also confirmed with *Xenopus* PrimPol_1–334_, which was also capable of reading through CPDs in an error-free manner (Supplementary Figure S1A). This TLS activity is similar to Pol η, a specialised TLS polymerase known to bypass CPDs in an error-free manner (21,22). As this read through of a CPD is only evident in the absence of the Zfn domain, it may be the case that PrimPol requires a conformational change or partner to assist in promoting this lesion bypass activity *in vivo*.

(6–4)PPs are a much more highly distorting UV lesion than CPDs. Pol η can incorporate bases opposite (6–4)PPs but incorporates an incorrect dG opposite the 3′ dT of the lesion and is unable to subsequently extend from this product (23). However, we previously reported that PrimPol exhibits the ability to incorporate opposite and extend through (6–4)PPs (4). Although PrimPol's TLS activity was retained when the Zfn was deleted, the nucleotide incorporation signature was altered (Figure 6B), again consistent with the proposed regulatory role of the Zfn domain. Wild-type PrimPol incorrectly incorporates thymine opposite the first dT of the (6–4)PP, whereas PrimPol_1–354_ incorporates dA or dC opposite this base. If dA nucleotide is incorporated opposite the first thymine of the (6–4)PP, PrimPol_1–354_ then incorporates either dC or dT. If the first nucleotide added is dC, it will then incorporate dA, dC or dT. To confirm whether this altered fidelity of nucleotide incorporation was orthologue-specific, we assayed the equivalent *Xenopus* PrimPol domain (XPrimPol_1–334_) and observed showed a similar fidelity to the human PrimPol polymerase domain (Supplementary Figure S1B).

**Figure 7.**
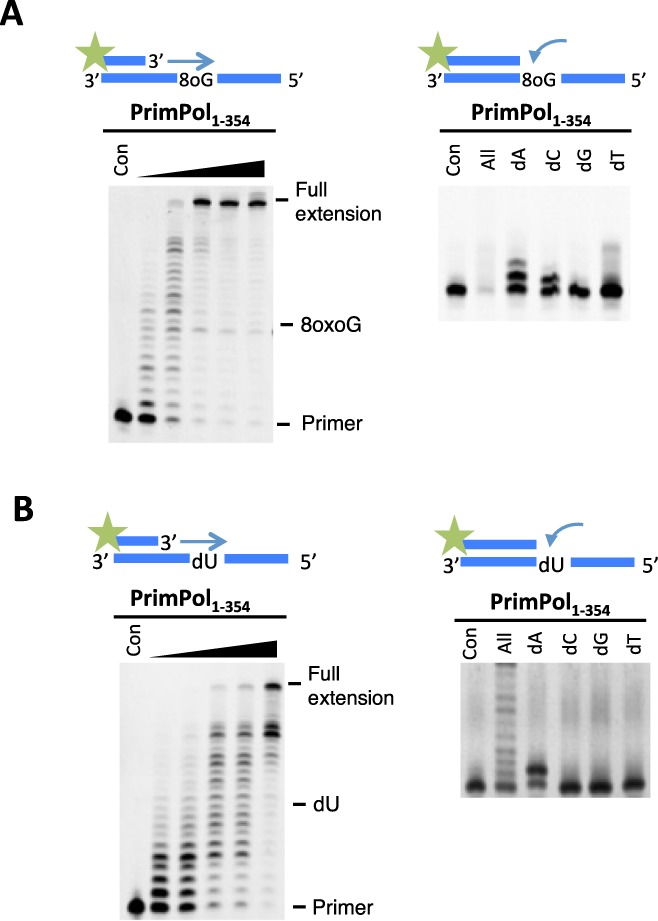
PrimPol_1–354_ can replicate through an 8-oxoguanine (8oxoG) lesion and a deoxyuracil (dU) base. (**A**) The polymerase domain exhibited minimal stalling opposite an 8oxoG lesion and extended fully to the end of the template. PrimPol incorporated an adenine or cytosine opposite the 8oxoG, which are the expected bases to be incorporated by Hoogsteen base pairing or Watson–Crick base pairing, respectively. (**B**) PrimPol_1–354_ efficiently synthesised through a templating dU site. PrimPol incorporates an adenine nucleotide opposite the dU lesion through Watson–Crick base pairing.

Next, we examined TLS bypass of an oxidative lesion, 8-oxoG. In common with wild-type enzyme, PrimPol_1–354_ can read through 8-oxoG lesions with minimal stalling and exhibited the ability to insert A and C opposite 8-oxoG lesions (Figure 7A), as previously observed for wild-type PrimPol (4). A similar TLS activity and fidelity for this lesion was also evident with XPrimPol_1–334_ (Supplementary Figure S2A). Cytosine in DNA is susceptible to spontaneous deamination to deoxyuracil (dU) *in vivo*, which can base pair to adenine, resulting in C→T transition mutations (10,11,24). Deoxyuracil is not recognised by, nor does it stall, eukaryotic TLS polymerases (10,13,14,25). Similarly, the polymerase domain of PrimPol does not stall when confronted with a dU base (Figure 7B). PrimPol incorporates an adenine opposite dU therefore, as one might expect, it reads the dU base as a thymine. This is in common with XPrimPol_1–334_ (Supplementary Figure S2B) and wild-type PrimPol (Supplementary Figure S3).

Finally, we examined TLS bypass of apurinic/apyrimidinic (AP sites) and thymine glycol (TG) lesions. PrimPol_1–354_ was unable to extend through either AP (Supplementary Figure S4A) or TG (Supplementary Figure S4B) lesions but there was some single nucleotide incorporated opposite the TG lesion. This insertion activity could potentially promote extension by another polymerase that is capable of extending following nucleotide insertion opposite the TG. XPrimPol_1–334_ was also unable to read through either of these lesions (Supplementary Figure S4). The inability of PrimPol_1–354_ to read through AP sites and TG lesions is also evident in wild-type PrimPol (4). This inability to extend through AP and TG sites, as well as other lesions, does not preclude the possibility that PrimPol reprimes following these lesions, allowing replicative polymerases to resume synthesis in a post-lesion fashion, see below.

### Human PrimPol is a distributive DNA polymerase

**Figure 8.**
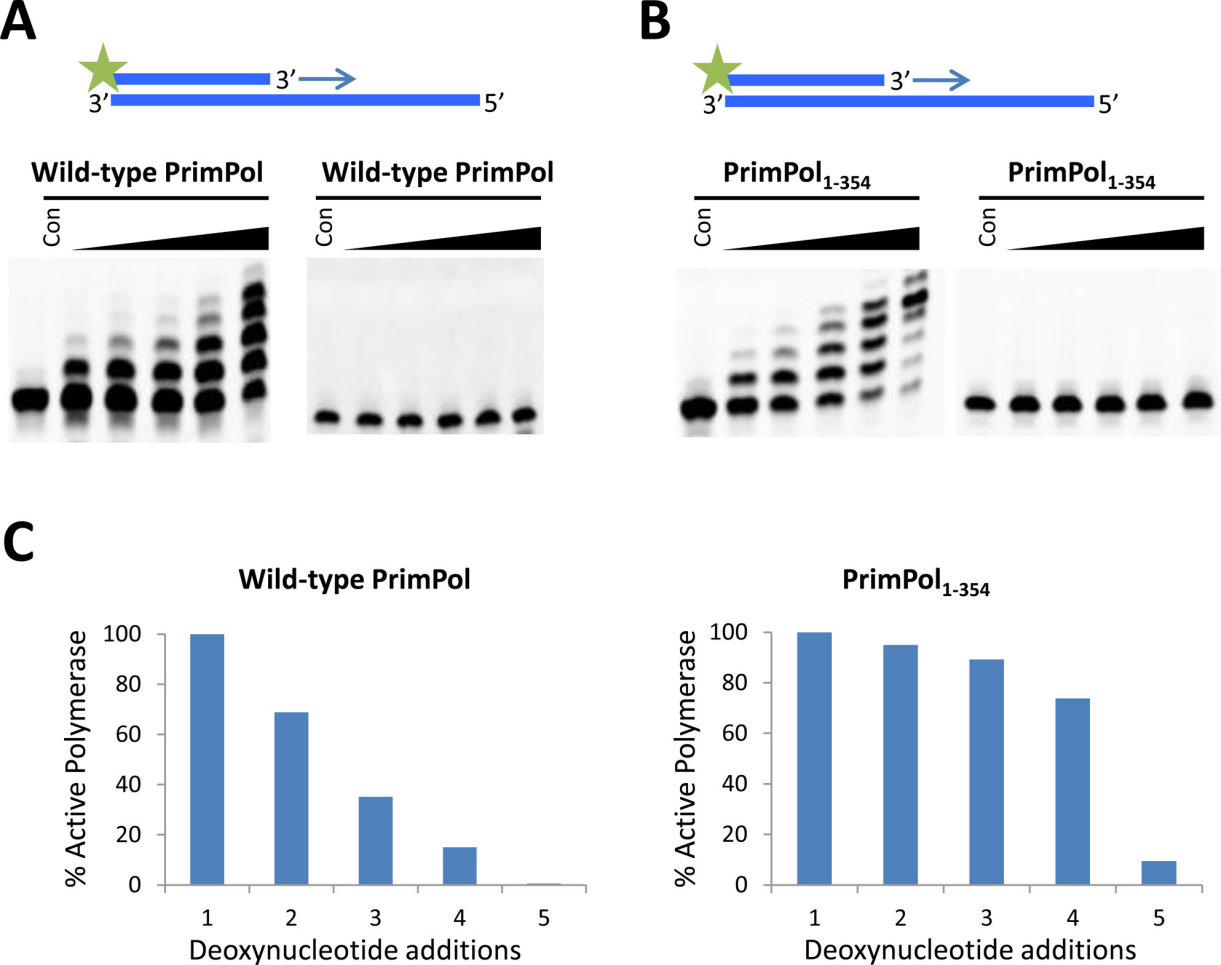
Processivity of the polymerase activity of PrimPol. PrimPol was pre-incubated for 30 min at 37°C with an undamaged DNA primer-template substrate to allow PrimPol to bind to the DNA. The reaction was initiated by the addition of dNTPs and an excess of sonicated herring sperm DNA (trap) and time points taken at 15, 30, 60, 120 and 360 s. (**A**) After 360 s, wild-type PrimPol incorporated up to 4 nucleotides opposite the template but a significant fraction of enzyme incorporated only 1, 2 or 3 nucleotides (left panel). To confirm that the trap prevents polymerase extending from a second template, the trap was also added into the pre-incubation mix with PrimPol and the DNA substrate. This reaction was supplemented with dNTPs and there is no extension (right panel), thus successfully exhibiting the effectiveness of the trap. (**B**) PrimPol_1–354_ also predominantly incorporates up to 4 nucleotides but there were fewer polymerases incorporating only 1, 2 or 3 nucleotides (left panel). The effectiveness of the trap was also successfully confirmed (right panel). (**C**) Percentage of PrimPol molecules incorporating at least *n* dNTPs for either full-length PrimPol or PrimPol_1–354_, calculated using Equation (1).

We next analysed the number of nucleotides incorporated by human PrimPol in a single association-dissociation event to measure the processivity of this polymerase. In order to determine the processivity of PrimPol, we performed primer extension assays in the presence of an excess of DNA (herring sperm), which acted as a ‘substrate trap' for polymerases that dissociate from the primer-template substrate. Using this approach, we identified that PrimPol inserts up to 4 nucleotides opposite an undamaged template, rarely inserting more than this (Figure 8A). When the Zfn domain was removed from PrimPol, the maximum number of nucleotides incorporated remained at ∼4 but the average number of nucleotides incorporated was higher, with fewer polymerases inserting 1–3 nucleotides (Figure 8B). We also carried out processivity assays in the presence of Taq polymerase to ensure that it was not the trap DNA that kept the polymerase from inserting more than 4 nucleotides. We observed that Taq polymerase possessed high processivity and incorporated nucleotides to the end of the template DNA (Supplementary Figure S5). Therefore, the Zfn appears to play a role in regulating the number of nucleotides that PrimPol inserts, possibly by stabilizing the interaction of the polymerase domain with the template, see the discussion.

### PrimPol's zinc finger domain is required for bypass of lesions *in vivo*

**Figure 9.**
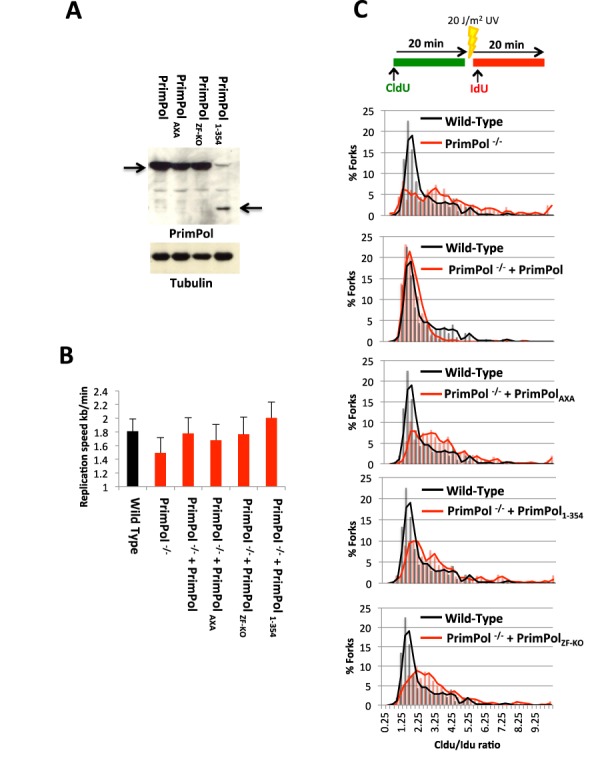
PrimPol's zinc finger domain is required for UV lesion bypass *in vivo.* (**A**) *PrimPol^−/−^* DT40 cells were complemented with variants of PrimPol and their *in vivo* expression confirmed by western blot. (**B**) Unperturbed replication fork rates were analysed by DNA fibre analysis using CldU and IdU labels for all the complemented cell types. The data represent the mean of three or more experiments with error bars showing the mean standard deviation. (**C**) DNA fork rate analysis was also carried out with after a 20 J/m^2^ pulse of UV, between CldU and IdU labels, to look for fork-stalling. Data is shown as the ratio of the two labels, Cldu, pre-UV : IdU, post UV label (*n* = 3). The black lines indicate the Cldu:IdU ratio of the two labels in the wild-type cells and the red lines indicate the ratios in cells complemented with the indicated PrimPol variants.

We previously reported that avian *PrimPol^−/−^* cells showed significantly decreased rates of replication fork progression (4). To evaluate the requirement for the Zfn domain of human PrimPol *in vivo*, we produced cell lines, in a DT40 *PrimPol^−/−^* knockout background (4), stably expressing human PrimPol, PrimPol_ZF-KO_, PrimPol_AXA_ (catalytically null) or PrimPol_1–354_ (Figure 9A). Using DNA fibre analysis, we confirmed that normal replication fork rates was restored by adding back human PrimPol, however this replication fork defect was not corrected by adding back the catalytically dead PrimPol_AXA_ mutant (Figure 9B). Next, we studied the replication fork speed in PrimPol_1–354_ and PrimPol_ZF-KO_ complemented clones and observed that both proteins were able to restore replication fork rates to near wild-type levels (Figure 9B). This suggests that the polymerase activity of PrimPol is largely responsible for the maintenance of fork progression in unperturbed replication. Indeed, PrimPol_1–354_ complemented cells showed even greater average replication fork rates compared to wild-type cells (Figure 9B), fitting with the superior polymerase activity observed *in vitro* (Figure 3A).

To examine the ability of the different PrimPol variants to support bypass of bulky lesions during replication *in vivo*, a large pulse of UV irradiation (20 J/m^2^) was introduced to cells between the two labelling steps to investigate fork stalling induced at UV lesions. PrimPol knockout cells show a pronounced stalling phenotype, indicated by a large increase in the ratio of the CldU to IdU ratios (4). Consistent with these previous findings, we observed complementation by adding back the wild-type enzyme. However, PrimPol_AXA_, PrimPol_1–354_ or PrimPol_ZF-KO_ were unable to correct this fork-stalling defect (Figure 9C). Therefore, although the polymerase extension activity of PrimPol is sufficient for maintenance of unperturbed replication, these data suggest that its primase activity is requisite for timely replication fork progression following the introduction of significant DNA damage.

## DISCUSSION

Recent studies have identified that PrimPol is a novel eukaryotic DNA polymerase capable of performing both priming and primer extension synthesis (4–7). It is likely that PrimPol was acquired early in eukaryotic evolution to play a significant role in lesion bypass during DNA replication. PrimPol-mediated damage tolerance appears to provide additional mechanisms that facilitate replication fork progression following stalling of replisomes at sites of DNA damage or secondary structure.

In this study, we have dissected the molecular activities associated with the major structural elements of human PrimPol (Table 1A) and determined the cellular requirements for these activities *in vivo*. PrimPol is both an active primase and polymerase with the capacity to perform TLS (Table 1B). An intact zinc finger is essential for PrimPol's primase activity *in vitro* (Figure 2), consistent with reports for a similar requirement for many diverse DNA primases (8,9,26,27). It has been proposed that this structural element is required for template recognition and regulation of primer length synthesis (28). Although the DNA polymerase activity of PrimPol appeared initially to be independent of the zinc finger domain, as a catalytically active domain lacking the zinc finger domain (PrimPol_1–354_) showed enhanced relative polymerase and processivity activities compared to the wild-type enzyme. Further studies revealed that the zinc finger modulates the extension activities and fidelity of PrimPol and therefore it should also be considered to be an important regulator of PrimPol's polymerase functions. PrimPol is a very distributive enzyme, catalysing up to four nucleotide incorporations during a single DNA substrate binding/polymerisation cycle *in vitro*. This distributive character of PrimPol is reminiscent of the DNA polymerase processivity reported for the Y-family TLS polymerases, such as Pol κ and η, which predominantly incorporate up to 6 or 7 nucleotides (16,29).

Although removal of the last 80 amino acids at the C-terminal region of human PrimPol (PrimPol_1–487_) did not abolish primase activity, which suggests that the C-terminus is not an absolute requirement for activity, it did reduce activity to some extent. Thermal denaturation studies have shown that there is a considerable increase in the stability of PrimPol upon removal of amino acids 488–560 (Supplementary Figure S6), suggesting that this region lacks discernible structure that would contribute to protein stability. Disordered regions of proteins often serve as scaffolds involved in protein–protein interactions and we speculate that this portion of human PrimPol might also act as an interaction interface. Indeed, it has been reported that the carboxyl-terminus of PrimPol mediates an interaction with the large subunit of human replication protein A (6).

In the presence of manganese, PrimPol appeared to be much more active than with magnesium. However, upon closer inspection, we discovered that the enzyme can produce polymeric primer extension products, even when only a single form of dNTP is present. Under these manganese-dependent conditions, PrimPol extends from a primer to form homopolymeric strands that are non-complementary and therefore unlikely to be annealed to the template strand. This suggests that caution should be taken when interpreting the results from studies using manganese as the resulting products may represent aberrant synthesis products rather than the expected template-dependent ones. Manganese has the effect of decreasing fidelity amongst polymerases (30,31). Whilst magnesium enforces tetrahedral geometry in the arrangement of its ligands, manganese will accommodate square, planar, tetrahedral and octahedral coordinations thus increasing the ability to accelerate the rate of reaction in substrates that are perhaps misaligned (32). The physiological implications of this template-independent primer extension activity of PrimPol in the presence of manganese remain unclear, as the cellular concentrations of manganese are low, suggesting this activity may be irrelevant *in vivo* or it may be limited to certain cellular compartments (e.g. mitochondria).

The translesion bypass activities associated with wild-type PrimPol and PrimPol_1–354_ are compared in Table 1B. As PrimPol can incorporate bases opposite and extend from highly distorting lesions, it is likely that the active site involved in production of phosphodiester bond is large and flexible to accommodate a range of lesions. The (6–4) photoproducts, in particular, are highly mutagenic due to the high degree of distortion they introduce into the DNA backbone. The helical bending at these lesions is estimated to be ∼44° and the distance between phosphorus atoms in the paired bases in the (6–4)PP is ∼6 Å, compared to the 7 Å average corresponding distance at other base-pairing steps (33). Therefore, it is probably unsurprising that PrimPol exhibits low fidelity when inserting bases opposite a (6–4)PP, as a polymerase that can accommodate this lesion would require a flexible active site. Unexpectedly, the polymerase domain of PrimPol, like Pol η, is capable of bypassing CPDs with high fidelity. This contrasts with full-length PrimPol, which is not capable of bypassing CPDs unless two adenines are inserted opposite the CPD (4,5). This result establishes that the active site of PrimPol has the capacity to bypass these lesions but this is constrained in the full-length enzyme. A conformation change may be required, potentially induced by binding partners, such as Replication Protein A (RPA), that alters the activity of PrimPol to allow it to bind to and bypass CPDs *in vivo.*

PrimPol is also confronted with a number of lesions as a result of oxidative stress in cells. These include AP sites and 8-oxoG lesions. Pol γ, the mitochondrial replicative polymerase, has the ability to bypass an 8-oxoG site but at a reduced rate, and cannot bypass AP sites (34,35). Conversely, PrimPol shows no hindrance to incorporate bases opposite 8-oxoG lesions and, although it cannot incorporate bases opposite an AP site, it has the capacity to re-prime post lesion. 8-oxoG is expected to pair with C through Watson–Crick base pairing in its regular *anti* conformation, but in its *syn* conformation it forms Hoogsteen base pairs with adenine (36). This ability to insert A, as well as C, opposite 8-oxoG is apparent in a number of polymerases but the addition of auxiliary proteins (e.g. RPA and PCNA) can dramatically increase the preference for adenine over cytosine, as is the case with Pol λ and η (36,37). It remains to be seen whether this is also the case with PrimPol. This ability to accommodate bulky lesions comes at the expense of fidelity and, therefore, PrimPol is a low fidelity polymerase that is likely to make limited, and perhaps, nonspecific contacts with the replicating base pair. Deoxyuracil is not recognised as a lesion by replicative polymerases and results in C:G → T:A transition mutations. Deoxyuracil is recognised by archaeal family B polymerases as a lesion and stalls replication to prevent mutation (38) but PrimPol does not have this recognition capacity and incorporates an adenine opposite a deoxyuracil lesion.

**Figure 10.**
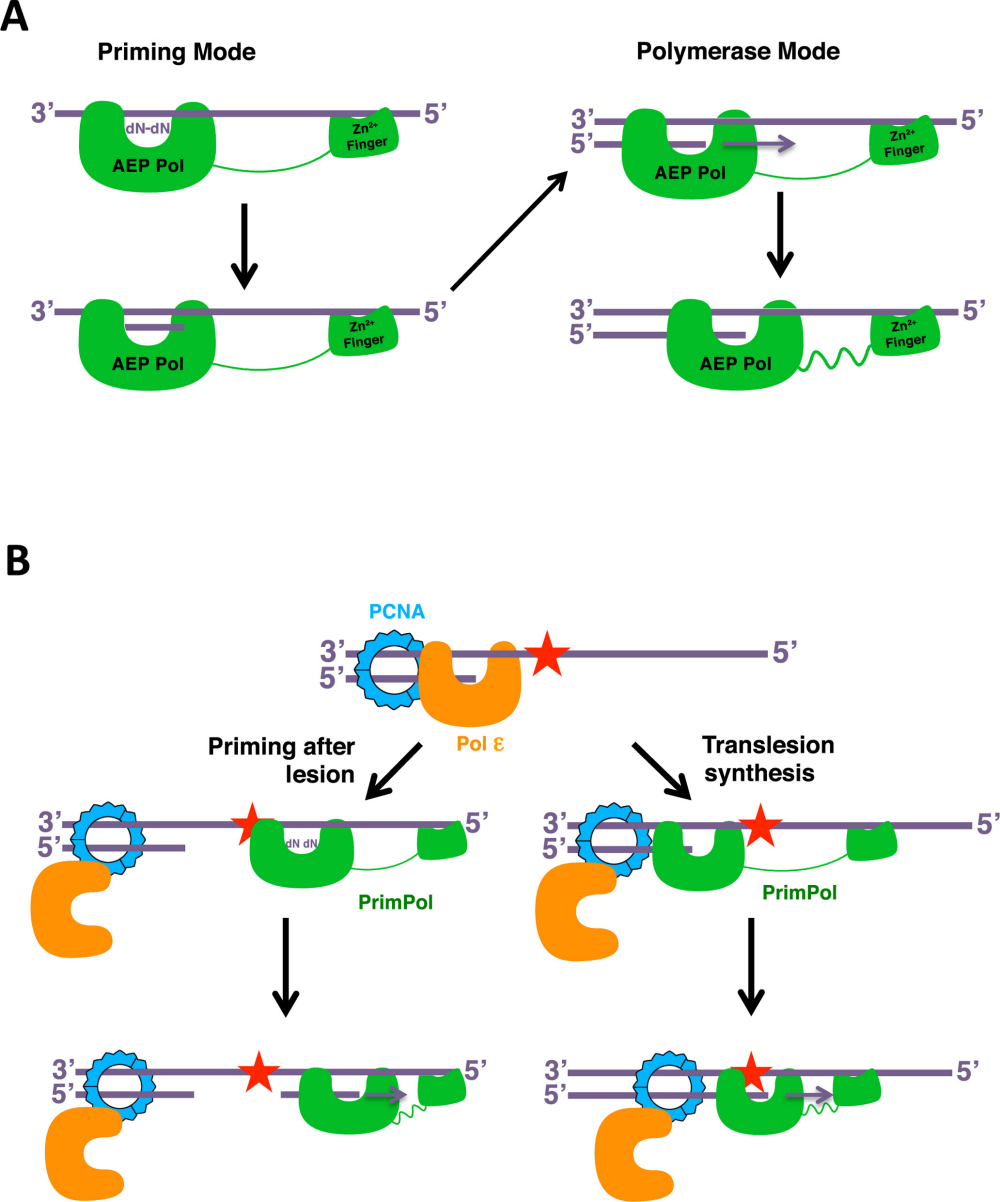
Models of the mechanisms of action of PrimPol. (**A**) PrimPol has both DNA primase and polymerase activities. The zinc finger of PrimPol stabilises the binding of the polymerase domain on single-stranded DNA. The AEP polymerase domain coordinates two adjacent deoxynucleotides opposite the single-stranded DNA template and the stability afforded to this complex by the Zfn binding allows synthesis of a dinucleotide. This dinucleotide can then be extended in a more processive way to form longer products. PrimPol can also bind primer-template junctions to catalyse more canonical primer extension reactions. Both the polymerase and primase activities are intrinsic to the AEP domain. Although the zinc finger is essential for maintaining less ‘stable' primer synthesis, it is also important for modulating the fidelity and processivity of the polymerase activities. This stabilizing influence of the Zfn may be particularly important for allowing productive synthesis during TLS bypass of distorting lesions and structures. We postulate that the primase and polymerase activities of PrimPol are not discrete activities but rather represent different synthesis modes performed under different DNA binding conditions. (**B**) PrimPol uses these activities to bypass DNA damage that blocks replicative polymerases. When replicative polymerases encounter a blocking lesion, they are displaced or regress from the site of damage. PrimPol can then access the template upstream of the lesion, where it can reprime following the lesion (left) or if it can access the primer-template junction it can carry out TLS (right). Once PrimPol-mediated bypass of the lesion has occurred, the replicative polymerase can then resume replication at the fork.

Canonical DNA polymerases share a common right-handed architecture containing fingers, palm and thumb subdomains. All of the three structural elements are clustered together providing accurate, highly structurally restrained positioning of the polymerase at the primer-template junction in order to promote the highest fidelity and processivity of DNA synthesis. Although this tight and specific binding cleft is perfectly suited for replication of long stretches of DNA, in some instances such structural arrangements can be disadvantageous. In particular, it limits the capacity of the replicative enzymes to tolerate abnormalities in the topology of DNA templates, such as secondary structures or DNA damage-induced distortions.The thumb subdomain of replicative polymerases interacts with the sugar-phosphate backbone of the DNA to coordinate the DNA. This subdomain is optimised for the highly accurate replication of DNA and is sensitive for structural anomalies in the in the DNA template. The predicted structure of PrimPol, in addition to the known structures of primases, suggests that this polymerase lacks structural elements corresponding to the ‘thumb’ domain that is present in the replicative polymerases that is implicated in processivity (39). The open planar predicted structure of this polymerase indicates a transient binding of the polymerase domain and this is consistent with other low fidelity polymerases, such as those of the Y-family of polymerases (40). The processivity data presented here suggests that the zinc finger domain of PrimPol regulates the fidelity of these polymerases. Whilst there is no reduction in the activity of the polymerase domain lacking the zinc finger, it actually appears more active, we propose that the presence of the zinc finger reduces the processivity of the enzyme to allow a slower, higher fidelity incorporation of complementary nucleotides. We postulate that whilst the AEP polymerase domain provides a platform to recognise the primer-template junction of the DNA substrate, the zinc finger binds the single-stranded region of the template strand to modulate binding and fidelity by the AEP domain (Figure 10A). Y-family polymerases have a highly truncated thumb domain and, notably, contain an additional functional module referred to as the polymerase associated domain (PAD). Interestingly, studies on TLS polymerases Dpo4 and Dbh point to a role for PAD in regulation of processivity and fidelity of these enzymes (41). This structural element not only binds ss DNA and provides an extended flexible platform coordinating the template strand during DNA synthesis on damaged DNA templates, but also modulates the activity of these TLS DNA polymerases. As part of an adaptation to perform TLS, we suggest that the zinc finger is functionally analogous to the PAD subdomain, but is spatially separated from the polymerase domain to allow greater freedom of binding of the enzyme to distorted DNA templates, such as those containing UV-induced lesions.

The activity most closely associated with the primase superfamily, namely primer synthesis, is considered to be somehow different from primer extension activities associated with canonical polymerases. However, primases should be considered to be polymerases that have evolved to catalyse a poorly processive extension reaction involving the synthesis of a dinucleotide. To do this, they have acquired the capacity to bind a single nucleotide as the ‘primer', which provides the 3′OH to initiate attack on a second template-bound nucleotide to form the first phosphodiester linkage. As Zfn binds single-stranded DNA and its absence negates primer synthesis, we propose that the Zfn acts in concert with the polymerase domain to stabilise the binding and configuration of the template, polymerase and nucleotides long enough to allow the production of the initial dinucleotide ‘primer' (Figure 10A). This product is subsequently extended in a more processive polymerisation mode. In replicative primases, the primase large subunit probably provides a similar role (12). In addition to primer synthesis, the zinc finger domain of PrimPol also plays additional roles in regulating the fidelity of these enzymes. By lowering the processivity of PrimPol, it appears to increase its fidelity, thus reducing its potential to be pro-mutagenic.

Finally, regarding the bypass of lesions during genome replication *in vivo*, this and previous work (4) suggest that both of PrimPol's priming and extension activities are requisite for lesion bypass and replication restart. When replicative DNA polymerases encounter a blocking lesion, they stall and PrimPol is recruited, possibly by RPA, to perform TLS or re-prime replication downstream of the lesion (Figure 10B). After production of a primer or polymerisation through the lesion, the replicative polymerase can then resume replication. PrimPol's extension activities appear to be PCNA-independent as no stimulation was observable in the presence of this protein (unpublished data). Although it has been reported that RPA can bind to and potentially recruit PrimPol to DNA, the mechanism by which it interfaces with stalled replisomes to restart fork progression remains to be established.

## SUPPLEMENTARY DATA

Supplementary Data are available at NAR Online.

SUPPLEMENTARY DATA
