# Supplementary material for: Molecular dissection of the domain architecture and catalytic activities of human PrimPol
Source: Nucleic Acids Res. 2014 Mar 20;42(9):5830–45. doi: 10.1093/nar/gku214 (PMC4027207; doi:10.1093/nar/gku214)
Supplement: SUPPLEMENTARY DATA [file supp_42_9_5830__index.html]

Molecular dissection of the domain architecture and catalytic activities of human PrimPol — Molecular dissection of the domain architecture and catalytic activities of human PrimPol — SUPPLEMENTARY DATA 

# Molecular dissection of the domain architecture and catalytic activities of human PrimPol

## SUPPLEMENTARY DATA

**Files in this Data Supplement:**

- SUPPLEMENTARY DATA
