# Supplementary material for: Molecular dissection of the domain architecture and catalytic activities of human PrimPol
Source: Nucleic Acids Res. 2014 Mar 20;42(9):5830–45. doi: 10.1093/nar/gku214 (PMC4027207; doi:10.1093/nar/gku214)
Supplement: SUPPLEMENTARY DATA [file supp_gku214_nar-03305-h-2013-File003.pdf]

Table S1

#	Primer	Sequence
1	1-354 PrimPol Forward Primer	5′-GTTTCTTCATATGAATAGAAAATGGGAAGCAAAACTG-3′
2	1-354 PrimPol Reverse Primer	5′-GTTTCTTGCGGCCGCGATACTGTTAAAATATCCAACC-3′
3	1-487 PrimPol Forward Primer	5′-GAAACTTAGAGCAATGAAACCCAGAATCC-3′
4	1-487 PrimPol Reverse Primer	5′-CATTGCTCTAAGTTTCATCTGCTTCATC-3′
5	Zinc Finger KO Forward Primer	5′-GTAAATATCGGTGGGCTGAAAACATTGGAAGAGCCGCTAAGAGTAATAATATAATG-3′
6	Zinc Finger KO Reverse Primer	5′-CTCTTAGCGGCTCTTCCAATGTTTTCAGCCCACCGATATTTACAAATATCATAAACCAGTAATTC-3′
7	372-560 PrimPol Forward Primer	5′-GTTTCTTCATATGCAGTGTTCTCCCTATCCTGAAGTTG-3′
8	372-560 PrimPol Reverse Primer	5′-GTTTCTTGCGGCCGCTTACTCTTGTAATACTTCTATAATTAGTTC-3′
9	X. tropicalis PrimPol Forward Primer	5′-GTTTCTTGGATCCATGAAAGCATGTGGAGGACTGGCAGATTATTATAG-3′
10	X. tropicalis PrimPol Reverse Primer	5′-GTTTCTTCTCGAGCTATTTGCTTGTTCGATAGTCAGGGTTGCTACATGTC-3′

Supplementary Table 1: Primers used in PCR in the production of recombinant PrimPol constructs. $\text{PrimPol}_{372\text{-}560/\text{ZF-KO}}$ was constructed by initially producing the $\text{PrimPol}_{\text{ZF-KO}}$ mutation variant, then using the 372-560 forward and reverse primers to isolate the required amino acids.

Table S2

#	Primer	Label	Sequence
1	Poly(dA)$_{60}$	5'-Biotin	5′-AAAAAAAAAAAAAAAAAAAAAAAAAAAAAAAAAAAAAAAAAAAAAAAAAAAAAAAAAAAA-3′
2	Poly(dC)$_{60}$	5'-Biotin	5′-CCCCCCCCCCCCCCCCCCCCCCCCCCCCCCCCCCCCCCCCCCCCCCCCCCCCCCCCCCCC-3′
3	Poly(dG)$_{60}$	5'-Biotin	5′-GGGGGGGGGGGGGGGGGGGGGGGGGGGGGGGGGGGGGGGGGGGGGGGGGGGGGGGGGGGG-3′
4	Poly(dT)$_{60}$	5'-Biotin	5′-TTTTTTTTTTTTTTTTTTTTTTTTTTTTTTTTTTTTTTTTTTTTTTTTTTTTTTTTTTTT-3′
5	HP-20 Primer	5'-Hex	5′-TGTCGTCTGTTCGGTCGTTC-3′
6	ND-50 Template	None	5′-CGCGCAGGGCGCACAACAGCCTTGAAGACCGAACGACCGAACAGACGACA-3′
7	HP-50 Primer	5'-Hex	5′-TGTCGTCTGTTCGGTCGTTCGGTCTTCAAGGCTGTTGTGCGCCCTGCGCG-3′
8	HP-27 Primer	5'-Hex	5′-TGTCGTCTGTTCGGTCGTTCGGTCTTC-3′
9	CPD Template	None	5′-CGCGCAGGGCGCACAACAGCCT=TGAAGACCGAACGACCGAACAGACGACA-3′
10	HP-28 Primer	5'-Hex	5′-TGTCGTCTGTTCGGTCGTTCGGTCTTCA-3′
11	6-4(PP) Template	None	5′- CTCGTCAGCATCT^TCATCATACAGTCAGTG -3′
12	HP-16 Primer	5'-Hex	5′-CACTGACTGTATGATG-3′
13	HP-17 Primer	5'-Hex	5′-CACTGACTGTATGATGT-3′
14	8-oxo-G Template	None	5′-CGCGCAGGGCGCACAACAGCC8-oxo-GTGAAGACCGAACGACCGAACAGACGACA-3′
15	AP Site Template	None	5′-CGCGCAGGGCGCACAACAGCCAPTGAAGACCGAACGACCGAACAGACGACA-3′
16	dUracil Template	None	5′-CGCGCAGGGCGCACAACAGCCUTGAAGACCGAACGACCGAACAGACGACA-3′
17	TG Template	None	5′-CGCGCAGGGCGCACAACAGCCTGTGAAGACCGAACGACCGAACAGACGACA-3′

Supplementary Table 2: DNA used in biochemical assays. For each substrate the labels, if any, are given. Any lesions in the DNA substrates are noted in red.

Table S3

Protein Construct	Protein Concentration (μM)	Zinc Concentration (μM)	Occupancy
PrimPol 372-560	80.0	64.34 ± 1.29	80.38%
PrimPol 372-560/ZF-KO	30.0	1.17 ± 0.05	3.90%
PrimPol 1-354	32.5	0.51 ± 0.023	1.57%

Supplementary Table 3: The PrimPol zinc finger domain ($PrimPol_{372\text{-}560}$) and zinc finger domain knockout ($PrimPol_{372\text{-}560/ZF\text{-}KO}$) were gel filtered in the absence of zinc to remove any zinc that was not chelated. They were subsequently tested for zinc concentration by inductively coupled plasma mass spectrometry (ICP-MS). The zinc finger domain exhibits zinc occupancy of 80.43%, whereas the zinc finger knockout only has occupancy of 3.9%, suggesting that the zinc chelation requires the intact amino acids that form the zinc finger motif (Cys-His-Cys-Cys, not Ala-Ala-Cys-Cys). The 1-354 polymerase domain was purified in the absence of zinc and was also subsequently tested for zinc occupancy, but only had 1.57% occupancy.

Figure S1

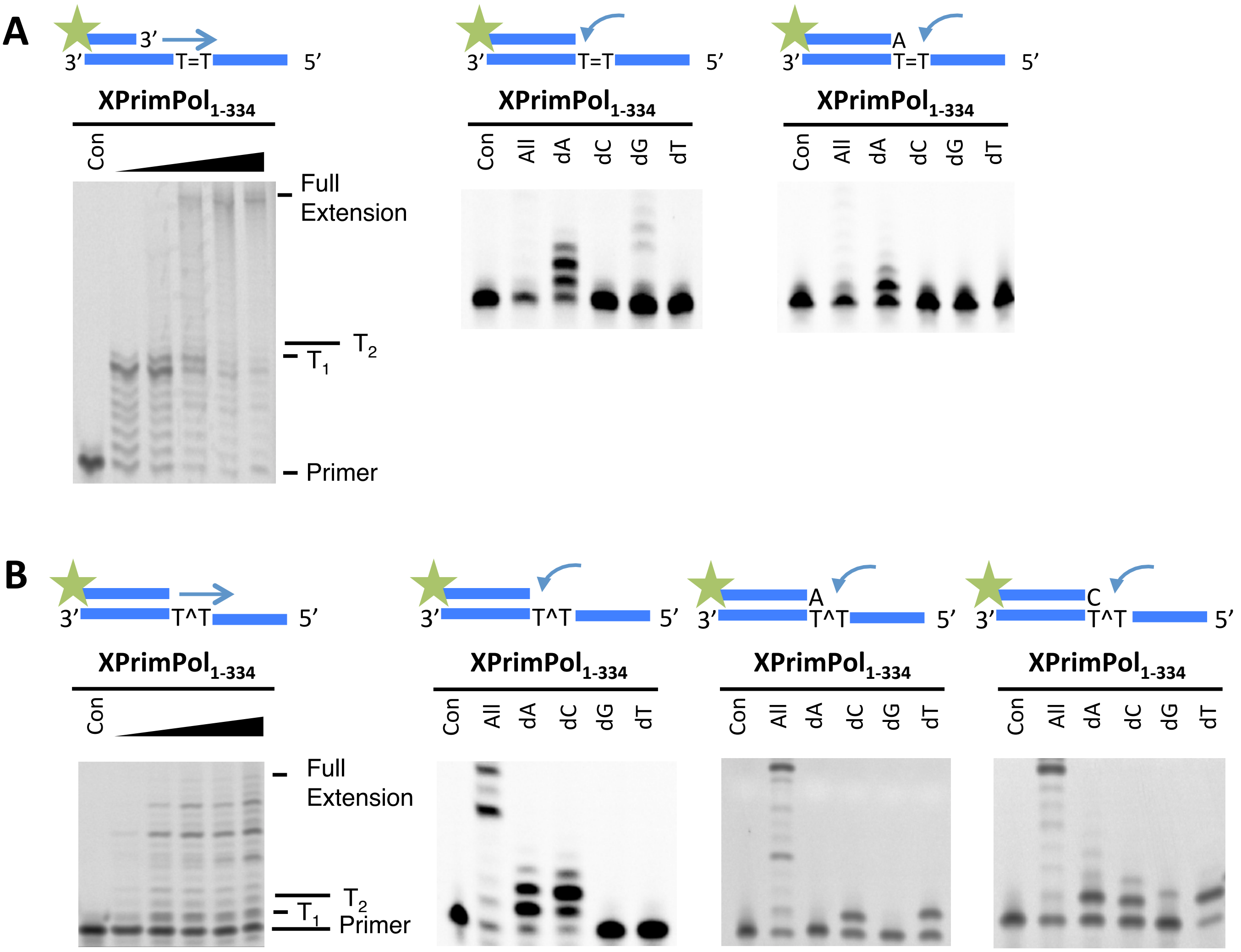

Figure S1: XPrimPol$_{1\text{-}334}$ can also replicate through CPD and 6-4 photoproduct (6-4(PP)) lesions caused by UV damage. **(A)** XPrimPol$_{1\text{-}334}$ was incubated with a primer-template substrate in which the template contained a CPD lesion downstream of the primer-template junction in the presence of dNTPs as in figure 5 with PrimPol$_{1\text{-}354}$. Similarly to PrimPol$_{1\text{-}354}$, XPrimPol$_{1\text{-}334}$ will extend up to a CPD, stall, then continue through the lesion. XPrimPol$_{1\text{-}334}$ will also incorporate two adenines opposite the first and second thymine of the CPD. **(B)** XPrimPol$_{1\text{-}334}$ will replicate through a 6-4(PP) and exhibits a similar fidelity to PrimPol$_{1\text{-}354.}$ XPrimPol$_{1\text{-}334}$ will incorporate an adenine or cytosine opposite the first thymine of the 6-4(PP). If it adds an adenine first, it will subsequently incorporate a cytosine or thymine opposite the second base of the 6-4(PP). If it adds a cytosine first, it will subsequently incorporate an adenine, cytosine or thymine nucleotide opposite the second base of the 6-4(PP).

Figure S2

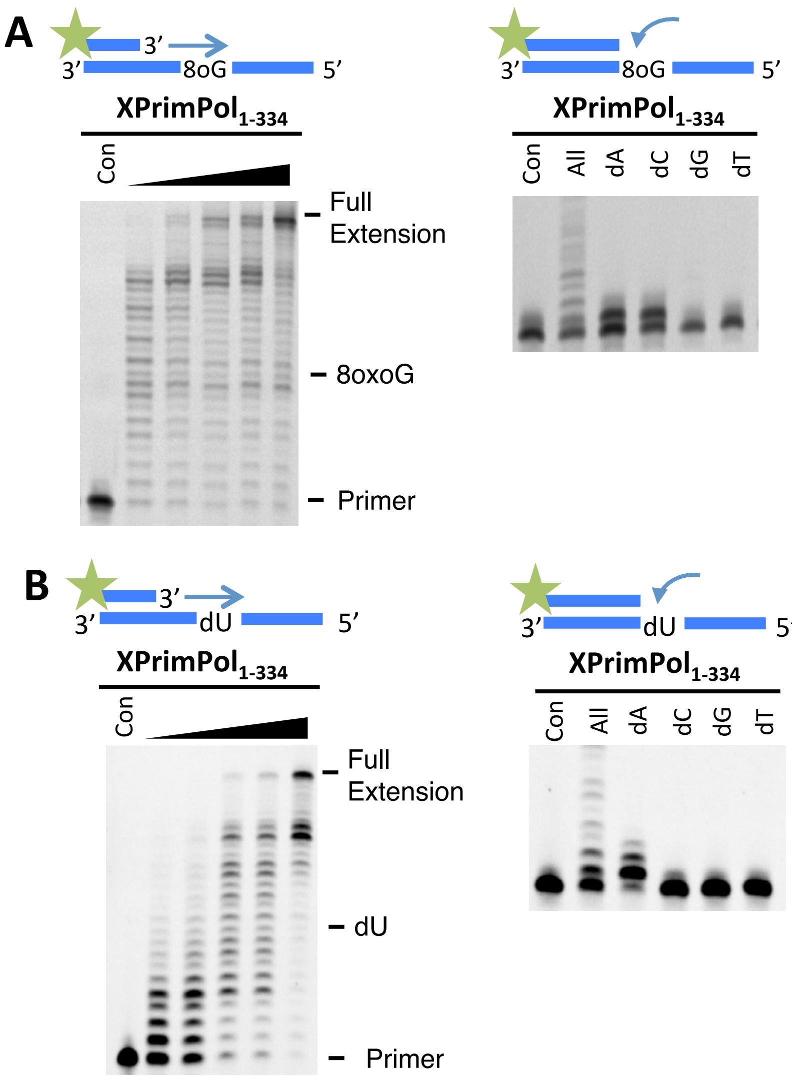

Figure S2: XPrimPol$_{1\text{-}334}$ can also replicate through an 8-oxoguanine (8oG) lesion and deoxyuracil (dU) **(A)** Similarly to PrimPol$_{1\text{-}354}$, the 8oG lesion is little obstacle to the polymerase domain of XPrimPol and efficiently replicates through this lesion. It will also incorporate an adenine or cytosine opposite the 8-oG lesion, through Watson-Crick or Hoogsteen base pairing respectively. **(B)** XPrimPol$_{1\text{-}334}$ can replicate through a dU base and will incorporate an adenine opposite the dU.

Figure S3

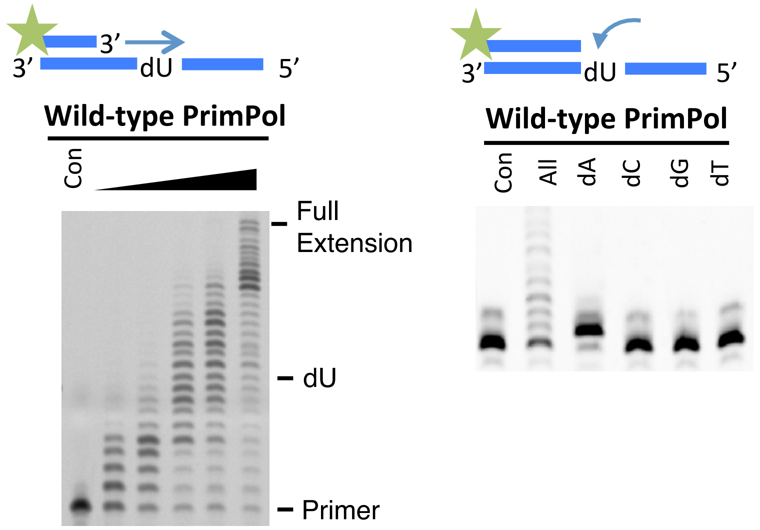

Figure S3: Wild-type Human PrimPol can replicate through a deoxyuracil (dU) base, which is often found in cells as caused by cytosine deamination. Similar to replicative polymerases, PrimPol incorporates an adenine base opposite a dU.

Figure S4

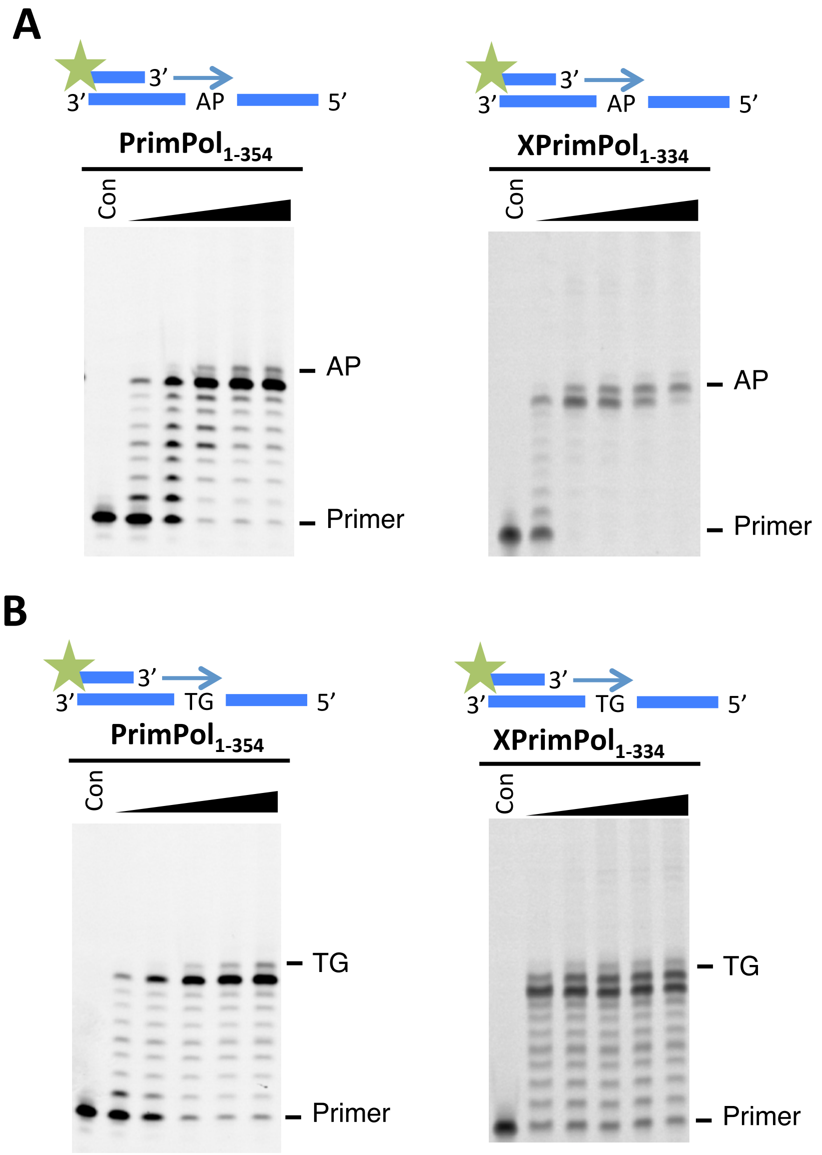

Figure S4: The polymerase domain of PrimPol is unable to read through an apyrimidinic/apurinic (AP) site or a thymine glycol (TG) lesion. **(A)** $\text{PrimPol}_{1\text{-}354}$ will incorporate nucleotides opposite a template to the AP site before stalling and there is some, but little, incorporation of a single base opposite the AP site. $\text{XPrimPol}_{1\text{-}334}$ stalls before an AP site, but can incorporate a single base opposite the AP site. **(B)** $\text{PrimPol}_{1\text{-}354}$ shows similar activity when confronted with a TG lesion, it will read up to the TG but not incorporate nucleotides past the damaged site. $\text{XPrimPol}_{1\text{-}334}$ also stalls before a TG lesion but can also incorporate a single base opposite the thymine glycol. $\text{XPrimPol}_{1\text{-}334}$ cannot then replicate from this incorporated base.

Figure S5

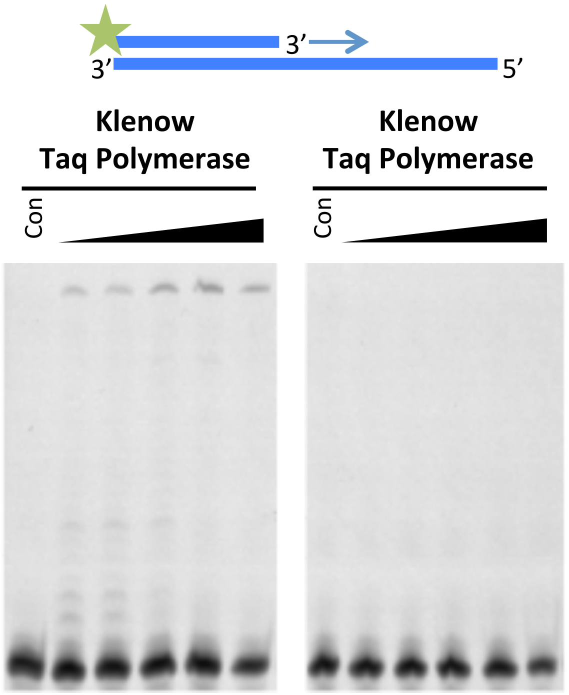

Figure S5: Processivity of the Klenow fragment of Taq polymerase. Klenow Taq polymerase was preincubated for 30 minutes at 37°C with an undamaged fluorescent DNA primer-template substrate to allow PrimPol to bind to the DNA. The reaction was initiated through the addition of dNTPs and an excess of sonicated herring sperm DNA trap and time points taken at 15 s, 30 s, 60 s, 120 s and 360 s. After 15 seconds Taq polymerase incorporates 30 nucleotides and is subsequently bound by the trap DNA. To confirm that the trap prevents polymerase extending from additional templates, the trap was also added into the preincubation mix with PrimPol and the DNA substrate. This reaction was supplemented with dNTPs and there is no extension, thus successfully exhibiting the effectiveness of the trap in the presence of the processive Taq polymerase.

Figure S6

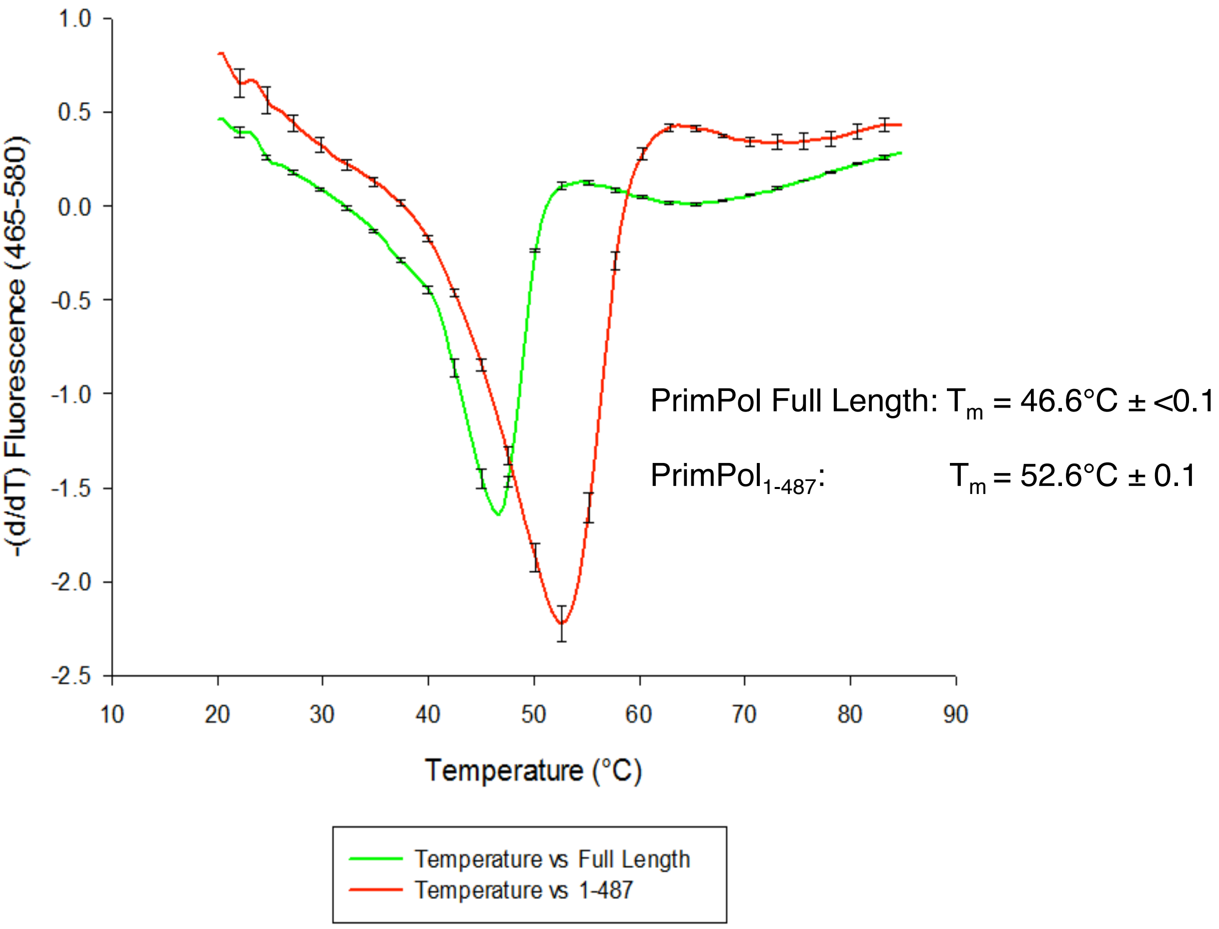

Figure S6: Thermal denaturation studies of Wild-type PrimPol and $PrimPol_{1\text{-}487}$. Full length PrimPol has a lower melting temperature (46.6°C ± <0.1) than $PrimPol_{1\text{-}487}$ (52.6°C ± 0.1), which suggests that the amino acids 487-560 at the C-terminus do not form a secondary structure and are instead unstructured regions of protein.
